# Mechanism driven design of trimer Ni_1_Sb_2_ site delivering superior hydrogenation selectivity to ethylene

**DOI:** 10.1038/s41467-022-33250-8

**Published:** 2022-09-21

**Authors:** Xiaohu Ge, Mingying Dou, Yueqiang Cao, Xi Liu, Qiang Yuwen, Jing Zhang, Gang Qian, Xueqing Gong, Xinggui Zhou, Liwei Chen, Weikang Yuan, Xuezhi Duan

**Affiliations:** 1 State Key Laboratory of Chemical Engineering, East China University of Science and Technology, 130 Meilong Road, Shanghai, 200237 China; 2 School of Chemistry and Chemical Engineering, In-situ Center for Physical Sciences, Frontiers Science Center for Transformative Molecules, Shanghai Jiao Tong University, Shanghai, 200240 China; 3 Key Laboratory for Advanced Materials, Centre for Computational Chemistry and Research Institute of Industrial Catalysis, East China University of Science and Technology, 130 Meilong Road, Shanghai, 200237 China

**Keywords:** Heterogeneous catalysis, Chemical engineering, Catalytic mechanisms

## Abstract

Mechanism driven catalyst design with atomically uniform ensemble sites is an important yet challenging issue in heterogeneous catalysis associated with breaking the activity-selectivity trade-off. Herein, a trimer Ni_1_Sb_2_ site in NiSb intermetallic featuring superior selectivity is elaborated for acetylene semi-hydrogenation via a theoretical guidance with a precise synthesis strategy. The trimer Ni_1_Sb_2_ site in NiSb intermetallic is predicted to endow acetylene reactant with an adequately but not excessively strong σ-adsorption mode while ethylene product with a weak π-adsorption one, where such compromise delivers higher ethylene formation rate. An in-situ trapping of molten Sb by Ni strategy is developed to realize the construction of Ni_1_Sb_2_ site in the intermetallic *P6*_*3*_*/mmc* NiSb catalysts. Such catalyst exhibits ethylene selectivity up to 93.2% at 100% of acetylene conversion, significantly prevailing over the referred Ni catalyst. These insights shed new lights on rational catalyst design by taming active sites to energetically match targeted reaction pathway.

## Abstract (web summary)

Designing atomically uniform ensemble sites for matching targeted reaction pathway is important yet challenging in heterogeneous catalysis. Here, the authors fabricate a trimer Ni_1_Sb_2_ site featuring superior selectivity for acetylene semi-hydrogenation via a mechanism-driven design strategy.

## Introduction

Design and fabrication of atomically dispersed active sites at supported catalysts for precisely tailoring configurations of key species to regulate the selectivity to the targeted product is of great importance in heterogeneous catalysis but remains a challenge^1–4^. Exemplified with acetylene semi-hydrogenation to ethylene in the purification and production of olefins, isolated metal sites, including single-atom metals anchored on supports^5–7^ and cationic metals confined in zeolites and MOFs^8–10^ have been proved to endow ethylene with π-adsorption mode toward higher product selectivity^9,11–13^, but usually leading to a trade-off between the activity and selectivity. In the case of PdZn alloy, it is pointed out that the two adjacent Pd sites isolated by guest Zn component give rise to the moderate σ-adsorption mode of acetylene reactant while the targeted π-adsorption mode of ethylene product^14^. An attempt is thus made to make full use of the guest metal bonding with an isolated host metal site, i.e., an isolated ensemble site with the spatially and energetically advantageous arrangement, aiming to break the activity-selectivity trade-off.

**Fig. 1:**
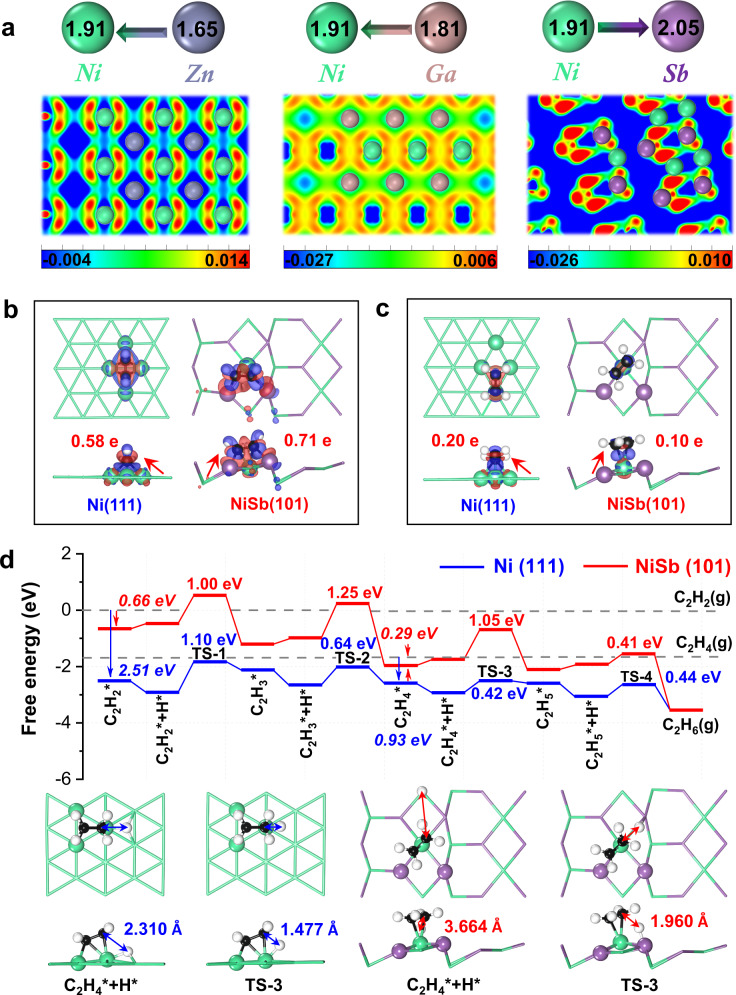
Theoretical insights into trimer Ni_1_Sb_2_ site. **a** Two-dimensional contours of the charge density difference of NiZn(110), NiGa(110), and NiSb(101) surfaces, where the units of the color bars are e·Bohr^−3^. Top and front views on the adsorption configurations of acetylene (**b**) and ethylene (**c**) adsorbed on Ni(111) and NiSb(101) surfaces, as well as the corresponding charge density distributions based on Bader charge analyses. The red and blue isosurfaces represent the accumulation and depletion of the electron density, respectively. The red arrows indicate the direction of charge transfer between the surfaces and the adsorbates. **d** Free energy profiles for sequential hydrogenation processes of acetylene to ethane on the Ni(111) and NiSb(101) surfaces, and the configurations of intermediates involved in the hydrogenation of C_2_H_4_. The blue and red arrows highlight the adsorption energies of C_2_H_2_ and C_2_H_4_ on the Ni(111) and NiSb(101) surfaces, respectively.

Zn and Ga with different electronegativities have been employed to fabricate the NiZn and NiGa intermetallic catalysts for the reaction^15,16^. The comparison of the performances of the NiZn and NiGa intermetallic catalysts demonstrates that modifying Ni sites with high-electronegativity metal would be more promising for enhancing acetylene semi-hydrogenation. Notably, the electronegativities of Zn and Ga are both lower than that of Ni, but the electronegativity of Ga is higher than that of Zn (1.81 vs 1.65), which gives rise to the lower electron density of the isolated Ni sites (Fig. 1a). In addition, introducing *p*-block element is revealed to feasibly modify the electronic properties of Ni sites via obvious *p*-*d* orbitals hybridization^17^. Inspired by these insights, employing a type of high-electronegativity p-block metal to isolate Ni sites and decrease their electron density, even to withdraw electrons from Ni sites, could facilitate the adsorption of electrophilic acetylene and desorption of nucleophilic ethylene, by means of which acetylene semi-hydrogenation to the target ethylene can be achieved.

To this end, we take advantage of the *p*-block features of guest Sb with high electronegativity to bond with the host Ni for the fabrication of trimer Ni_1_Sb_2_ site, atomically distributed on the intermetallic NiSb, for boosting acetylene semi-hydrogenation. The unique features of Sb contribute to electron-defect Ni sites isolated by electron-rich Sb ones (Fig. 1a). Such ensemble site enables both moderate σ-adsorption of electrophilic acetylene and weak π-adsorption of nucleophilic ethylene, as demonstrated by systematic density functional theory calculations and temperature-programmed desorption experiments. An in situ trapping of molten Sb by Ni strategy was developed to realize the fabrication of the trimer Ni_1_Sb_2_ site in the NiSb intermetallic catalyst. The unique structural features of the NiSb catalyst were thoroughly identified by multiple techniques, including atomic-resolution electron microscopy and X-ray absorption spectroscopy. As predicted by the theoretical calculations, the NiSb catalyst featured with the trimer site exhibited an excellent ethylene selectivity, i.e., up to 93.2% at 100% of acetylene conversion, which delivers a superior formation rate of ethylene compared with the referred Ni catalyst.

## Results and discussion

### Theoretical design of trimer Ni_1_Sb_2_ site

We resort to DFT calculations for exploring the guest Sb bonding with the isolated Ni site, having a spatially and energetically advantageous arrangement, to break the activity-selectivity trade-off. The introduction of antimony remarkably changes the crystal structure, as revealed by the Wulff construction crystals in Supplementary Fig. 1 and Supplementary Tables 1, 2. The Ni(111) and NiSb(101) surfaces, schematically shown in Supplementary Fig. 2, are indicated as the most exposed surfaces for Ni and NiSb, respectively. Considering that the most exposed surface plays a major role in acetylene hydrogenation, the Ni(111) and NiSb(101) surfaces were thus selected for DFT calculations. The *P6*_*3*_*/mmc* NiSb intermetallic phase, exposing the atomically distributed and ensemble Ni_1_Sb_2_ sites, and the referenced Ni face-center cubic phase were modeled (Supplementary Fig. 2), which are rationalized by the good accordance of XRD simulations with the previous experiments^18–21^. On the typical and thermodynamically stable NiSb(101) surface (Supplementary Tables 5, 6), the C_2_H_2_ reactant is found to thermodynamically bind with the trimer Ni_1_Sb_2_ site via a σ-adsorption mode with the adsorption-free energy of −0.66 eV, while the C_2_H_4_ product with the single Ni site via a π-adsorption one with that of −0.29 eV. In contrast, on the Ni(111) surface, both the C_2_H_2_ and C_2_H_4_ prefer to σ-adsorption modes strongly binding with four- and three-hollow Ni sites (i.e., −2.51 and −0.93 eV), respectively.

The crucial role of the guest Sb was further elucidated by electronic structures analysis compared with previously reported Ga for isolating Ni sites in NiGa intermetallics^16^ (Fig. 1a and Supplementary Fig. 3). Bader charge analyses reveal less electron transfer associated with the adsorption of C_2_H_4_ but unexpectedly more with that of C_2_H_2_ on the trimer site as compared to the analysis for those on the Ni surface (Fig. 1b, c). Differently, the electron transfer from the NiGa surface to the adsorbed acetylene molecule is lower than that from the Ni surface (0.16 e vs 0.58 e), which is in consistent with the lower adsorption energy on the NiGa surface. These could be caused by the electronic interaction between C atoms of acetylene and Sb atoms of trimer Ni_1_Sb_2_ site in the σ-adsorption configuration, as suggested by the hybridization of C *2p* with Sb *5p* orbitals (Supplementary Fig. 3). The C *2p* orbital DOS profiles of acetylene are similar on the NiGa and Ni surfaces, and the C *2p* orbital is mainly hybridized with the Ni *3d* orbital with the absence of obviously hybridized to the Ga *4p* orbital. These results confirm that the Sb with higher electronegativity than that of Ni is more promising to be employed to isolate the Ni sites toward enhanced acetylene semi-hydrogenation.

The hydrogenation process of acetylene via the Horiuti–Polanyi mechanism^22–24^ on the trimer site was subsequently studied by theoretical calculations. On the trimer Ni_1_Sb_2_ site, the adsorbed C_2_H_2_^*^ is sequentially hydrogenated to C_2_H_3_^*^ and C_2_H_4_^*^ with free energy barriers of 1.00 and 1.25 eV (Fig. 1d and Supplementary Fig. 5), respectively. Notably, the hydrogenation of the formed C_2_H_4_^*^ needs to overcome a free energy barrier of 1.05 eV on the trimer Ni_1_Sb_2_ site, which is higher than the desorption free energy of C_2_H_4_^*^. In contrast, the hydrogenation of C_2_H_4_^*^ formed from two-step hydrogenations of C_2_H_2_^*^ with free energy barriers of 1.10 and 0.64 eV is more facile than the desorption process on the referenced Ni sites, as suggested by the 0.42 eV of hydrogenation free energy barrier against the 0.93 eV of the desorption free energy (Fig. 1d and Supplementary Fig. 4). The competitiveness between the hydrogenation of C_2_H_4_^*^ to C_2_H_5_^*^ and the desorption of C_2_H_4_^*^ determines the ethylene selectivity^16,24^. These energetic scenarios strongly indicate that the hydrogenation of C_2_H_4_^*^ is suppressed on the trimer site while favorable on the referenced Ni site, which are further traced to be the unfavorable configuration of the initial and transition states on the trimer Ni_1_Sb_2_ site. The distances between the H^*^ and C atom of π-adsorbed C_2_H_4_ measured for the initial and transition states on the Ni_1_Sb_2_ site are 3.664 and 1.960 Å (Fig. 1d and Supplementary Fig. 5), respectively, which are remarkably longer than those measured for the configurations on the Ni site, evidencing the thermodynamically unstable configuration of C_2_H_4_^*^ and H^*^ on the NiSb(101) surface. These results clearly demonstrate that the desorption process of C_2_H_4_ on the trimer site with π-adsorption is more facile than its over-hydrogenation, and thus the formation of targeted ethylene would be promoted. Previous studies have well shown that the moderate σ-adsorption mode of acetylene is more favorable for hydrogenation activity than the π-adsorption one^5–7,14^. Thus, the moderate σ-adsorption of acetylene and a weak π-adsorption of ethylene on the trimer Ni_1_Sb_2_ site would deliver the possibility of breaking the trade-off between activity and selectivity for the reaction.

Furthermore, we also investigated the hydrogenation of acetylene on the NiSb(102) surface (Supplementary Figs. 6, 7), which is the secondly mostly exposed surface as suggested by the Wulff constructions (Supplementary Fig. 1). The adsorption configuration of C_2_H_2_ on the NiSb(102) surface is similar to that on the NiSb(101) surface, binding to the trimer Ni_1_Sb_2_ site via the σ-adsorption mode with an adsorption-free energy of −0.89 eV (Supplementary Table 7). The C_2_H_4_ prefers to adsorb on the isolated Ni site with adsorption-free energy of −0.36 eV (Supplementary Table 8). On the trimer Ni_1_Sb_2_ site of the NiSb(102) surface, the calculated free energy barriers for the two-step hydrogenation of C_2_H_2_^*^ to C_2_H_4_^*^ are 0.63 and 1.31 eV (Supplementary Figs. 6, 7), respectively. The desorption free energy of C_2_H_4_^*^ on the NiSb(102) surface is 0.36 eV, which is lower than the free energy barrier for the further hydrogenation to C_2_H_5_^*^ (1.10 eV), indicating the ethylene product prefers to desorb from the surface rather than be hydrogenated. Thus, it can be concluded that the trimer Ni_1_Sb_2_ site on the NiSb(102) surface also exhibits good ethylene selectivity, which is similar to that on the NiSb(101) surface.

In addition, the dissociation and diffusion of hydrogen were also evaluated on the Ni(111), NiSb(101), and NiSb(102) surfaces. The hydrogen molecule prefers to adsorb on the top site of Ni(111) surface and subsequently dissociate into two H atoms with an energy barrier of 0.02 eV and an exothermic energy of −0.98 eV (Supplementary Fig. 10). These results indicate that the dissociation of hydrogen is facile on the Ni(111) surface. In contrast, the hydrogen molecule adsorbed on isolated Ni sites of NiSb(101) and NiSb(102) surfaces dissociate with energy barriers of 0.83 and 0.80 eV with endothermic energy of 0.10 and 0.07 eV, respectively (Supplementary Figs. 11, 12). These results reveal that the H_2_ dissociation is less facile on the NiSb(101) and NiSb(102) surfaces with isolated Ni sites than that on the Ni(111) surface, which could decrease the coverage of hydrogen on the NiSb(101) and NiSb(102) surfaces for hydrogenation of formed ethylene to undesirable ethane. Hydrogen diffusions in the presence of C_2_ species were further simulated to evaluate the ease of hydrogen diffusion to the nearby C_2_ species for subsequent hydrogenation. As shown in Supplementary Figs. 13–16, the energy barriers of hydrogen diffusion on the Ni(111) surface are in the range of 0.15–0.24 eV during the hydrogenation pathways, while those on the NiSb(101) and NiSb(102) surfaces are in the range of 0.59–0.66 eV and 0.65–0.71 eV, respectively. These results suggest that the diffusion of hydrogen atoms on the Ni(111) surface is more facile than those on the NiSb(101) and NiSb(102) ones, which could be caused by the elongated Ni-Ni distances on the latter ones. Such easily activated and movable hydrogen atoms on Ni(111) surface could favor the hydrogenation of surface C_2_ species, including the formed ethylene on the surface, and thus lead to the suppressed formation of ethylene while promoted one of ethane.

Acetylene hydrogenation was also investigated on the minorly exposed NiSb(100) in which the distance between the nearest Ni sites is 2.643 Å, much shorter than those in NiSb(101) and NiSb(102) surfaces (Supplementary Fig. 2). The results are summarized in Supplementary Tables 9, 10 and Supplementary Figs. 8,9. The desorption energy for ethylene (0.91 eV) is higher than the hydrogenation barrier (0.78 eV) on the NiSb(100) surface, suggesting that the hydrogenation of ethylene formed on the surface is more facile than ethylene desorption. Thus, the predominantly exposed surface NiSb(101) and NiSb(102) surfaces featuring trimer Ni_1_Sb_2_ sites dominate the semi-hydrogenation to ethylene, while the minorly exposed NiSb(100) surface leads to the over-hydrogenation to ethane. These results highlight the significance of trimer Ni_1_Sb_2_ sites for acetylene semi-hydrogenation.

### Synthesis and structural characterizations

**Fig. 2:**
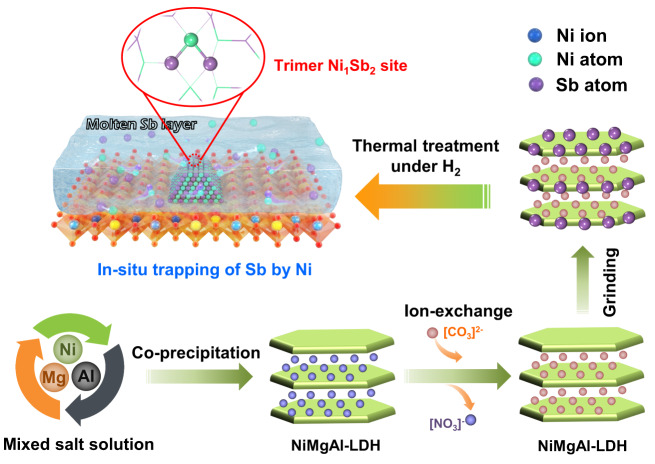
Synthesis strategy for Ni_1_Sb_2_ sites. Schematic illustration for the construction of the trimer Ni_1_Sb_2_ site in the NiSb intermetallic structure via an in situ trapping of molten Sb by Ni strategy facilitated by thermal treatment under hydrogen from the Ni/Mg/Al-LDHs precursor.

**Fig. 3:**
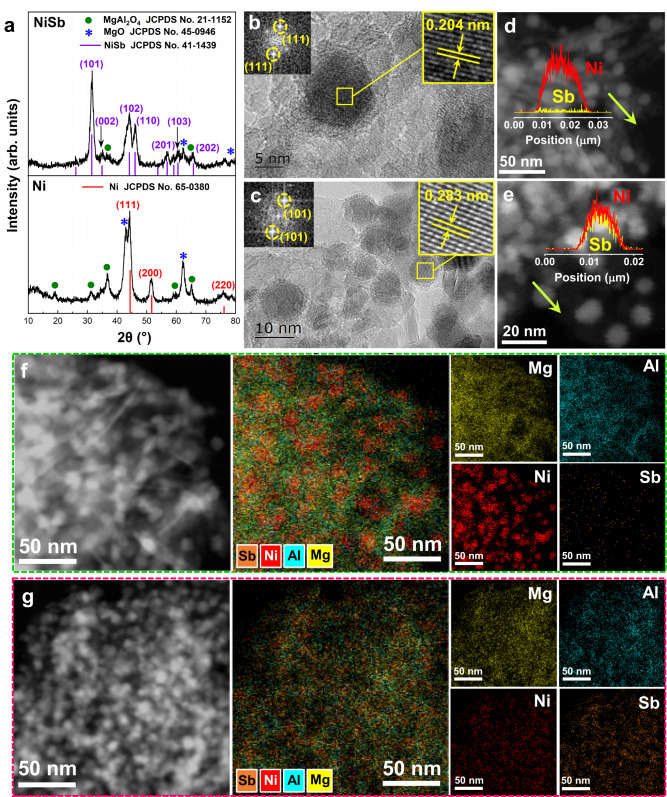
Microstructure and morphology of NiSb intermetallic catalyst. **a** XRD patterns of the synthesized Ni and NiSb catalysts. Typical HRTEM images and corresponding FFT patterns of **b** Ni and **c** NiSb catalysts. HAADF-STEM images of **d** Ni and **e** NiSb catalysts as well as the corresponding EDS line-scanning profiles. HAADF-STEM EDS mapping analyses of **f** Ni and **g** NiSb catalysts.

Followed by the theoretical insights, the trimer Ni_1_Sb_2_ site was subsequently realized in the NiSb intermetallic catalysts synthesized by an in situ trapping strategy (Fig. 2). The bulk Sb powder was grinded with the Ni/Mg/Al-LDHs precursors prepared by a co-precipitation method to obtain the Sb-containing LDHs, which was further treated at 900 °C under hydrogen. At such a high temperature, bulk Sb was melted into a molten and movable state. Simultaneously, the high temperature led to the reduction of Ni atoms from Ni ions contained in the LDHs and further ex-solution from the LDH. The strong NiSb metallic bond interaction favors the free Ni atoms to bond with the Sb atoms to form NiSb motifs, which thermodynamically assemble to form the nanoparticles featured with a stable NiSb intermetallic structure. The crystal structure of the synthesized NiSb catalyst was identified by XRD measurements (Fig. 3a). The diffraction peaks at 36.8°, 59.3°, and 65.2° are corresponding to the (311), (511), and (440) planes of MgAl_2_O_4_ (JCPDS No. 21–1152), and the two diffraction peaks at 42.9° and 62.3° are assigned to the (200) and (220) planes of MgO (JCPDS No. 45–0946), respectively. In addition to these diffraction peaks of the support, the XRD pattern of the NiSb catalyst exhibits typical diffraction peaks at 31.5°, 43.9°, and 46.1° assigned to the (101), (102), and (110) planes of hexagonal NiSb intermetallic phase (JCPDS No. 41–1439), respectively. These results imply the successful formation of the NiSb intermetallic phase in the NiSb catalyst via the strategy. In contrast, representative diffraction peaks at 44.3°, 51.7°, and 76.1° ascribed to the (111), (200), and (220) planes of face-centered cubic Ni phase (JCPDS No. 65–0380), respectively, are observed from the XRD pattern of the Ni catalyst.

To verify the trapping roles of Ni species in LDHs, a referred LDHs without Ni species was synthesized and then grinded mechanically with Sb powder. After thermal treatment at 900 °C under hydrogen, the crystal structure of the as-obtained sample was comparatively characterized by XRD, which shows the absence of diffraction peaks corresponding to Sb phases (Supplementary Fig. 17). Furthermore, the determined content of Sb in this referred catalyst is close to zero (Supplementary Table 11). These results clearly indicate that the movable and molten Sb cannot be trapped by the LDHs without Ni species and finally vaporized during the thermal treatment^25^, and thus could detach from the solid sample. Further increasing the amount of Sb in the 2-NiSb catalyst, where 2 denotes the nominal ratio of Sb/Ni, also gives rise to the formation of the NiSb intermetallic phase by this trapping strategy rather than the NiSb_2_ intermetallic phase (Supplementary Fig. 17 and Figs. 23,  26), probably owing to the NiSb metallic bond interaction with a ratio of 1:1 during the trapping process. The actual atomic ratio of Sb/Ni is determined to be 1.07 (Supplementary Table 11), close to the nominal one of the NiSb intermetallic phase, evidencing that the extra Sb could be vaporized and then detach from the solid sample^25^. The evaporation of Sb in the precursor of the 2-NiSb during the thermal process was further confirmed by thermogravimetric analysis in Supplementary Fig. 18 and Fig. 19.

High-resolution transmission electron microscopy (HRTEM) images and high-angle annular dark-field scanning transmission electron microscopy (HAADF-STEM) measurements were further carried out to identify microstructural features of monometallic Ni and NiSb catalysts. Supplementary Fig. 20 shows the typical HAADF-STEM images of the Ni and NiSb catalysts, where the nanoparticles are uniformly distributed with similar average particle sizes. The typical HRTEM images with the corresponding fast Fourier transform (FFT) patterns of Ni and NiSb catalysts demonstrate continuous lattice fringes with the spacings of 0.204 and 0.283 nm (Fig. 3b, c), which are assigned to the interplanar spacings of the (111) plane of face-centered cubic Ni and the (101) plane of the hexagonal NiSb intermetallics, respectively. Furthermore, the energy-dispersive X-ray spectroscopy (EDS) line-scanning analyses for single nanoparticle indicate that Ni and Sb are homogeneously distributed over the nanoparticles with the atomic ratio of 1:1 (Fig. 3e and Supplementary Fig. 21), as compared with those of Ni catalyst (Fig. 3d and Supplementary Fig. 22). Similarly, the EDS mapping analyses for the Ni catalyst exhibit the uniform distribution of Ni within the monometallic nanoparticles with the absence of Sb (Fig. 3f and Supplementary Fig. 24), while those of the NiSb catalyst demonstrate that Ni and Sb species are homogeneously distributed in bimetallic nanoparticles (Fig. 3g and Supplementary Fig. 25). All these results point to the successful fabrication of the NiSb intermetallic phase.

**Fig. 4:**
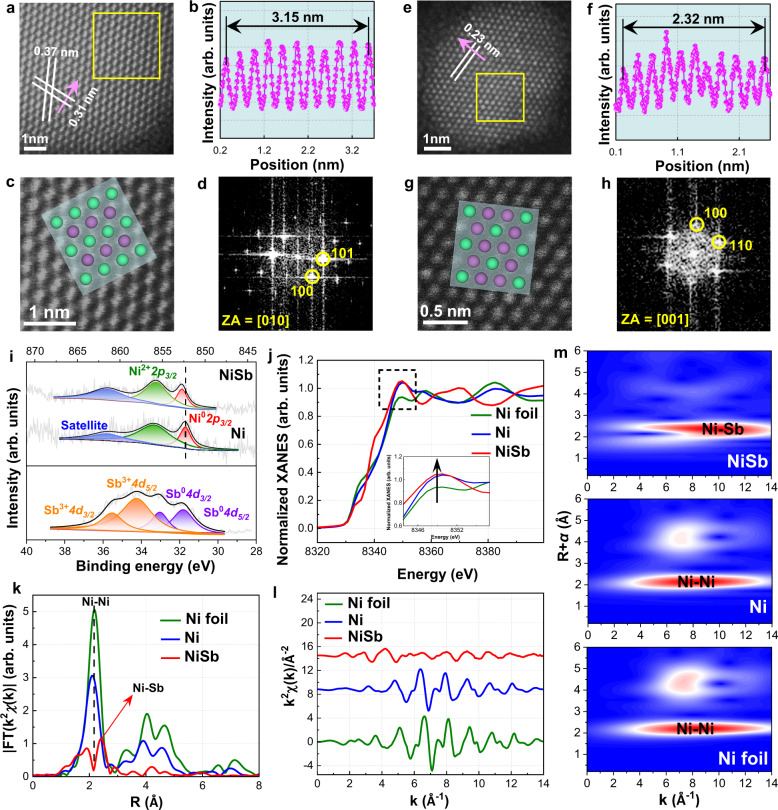
Atomic structures of NiSb intermetallic catalyst. **a**, **e** Representative AC-HAADF-STEM images of NiSb catalyst along with different zone axes. **b**, **f** Line intensity profiles along direct indicated by the violet arrows in **a** and **e**. **c**, **g** Enlarged views of the areas marked by the yellow rectangles in **a** and e as well as the corresponding crystal models along with [010] and [001] zones, respectively. **d**, **h** The FFT patterns of the NiSb catalyst. **i** Ni *2p* and Sb *4d* XPS spectra of the NiSb catalyst. **j** Normalized XANES spectra and **k** k^2^-weighted Fourier transform (FT) spectra at the Ni K-edge of the Ni foil, Ni, and NiSb catalysts. **l** The EXAFS oscillation functions and **m** WT-EXAFS contours at the Ni K-edge of Ni foil, Ni, and NiSb catalysts.

Aberration-corrected HAADF-STEM (AC-HAADF-STEM) technique was employed for visualizing the atomic structure of the NiSb catalyst. Figure 4a shows the typical AC-HAADF-STEM image of the NiSb catalyst, where distinct lattice fringes are clearly observed. The integrated pixel intensity profile acquired along with the violet arrow in Fig. 4b reveals that the average spacing along this direction is 0.31 nm assigned to the (101) plane of intermetallic NiSb. Similarly, the average spacing of another lattice fringe is determined to be 0.37 nm corresponding to the (100) plane of the NiSb intermetallic phase. Based on the enlarged image of atomic distribution in Fig. 4c, the corresponding FFT pattern is obtained to derivate the zone axis (Fig. 4d). The atomic distribution predicted by the crystal structural models along with the determined [010] zone axis is well consistent with that visualized by AC-HAADF-STEM in Fig. 4c. To further check the formation of NiSb intermetallic phase in NiSb catalyst, another nanoparticle in a different area is also randomly selected to characterize by AC-HAADF-STEM (Fig. 4e). Similarly, ordering atomic arrangement with distinct lattice fringe is clearly seen in Fig. 4f, and the integrated pixel intensity profile shows that the average spacing of such lattice fringe is determined to be 0.23 nm assigned to the (110) plane of NiSb intermetallic phase. The obtained FFT pattern according to the enlarged image provides the zone axis (Fig. 4g, h), based on which the predicted arrangement from the ideal NiSb crystal structure agrees well with the observed one. Similar results are also seen with other particles shown in Supplementary Figs. 27,  28. These unequivocally demonstrate the ordering atomic distribution of the NiSb intermetallic phase in the synthesized NiSb catalyst.

The formation of the NiSb intermetallic structure would deliver remarkable electronic interaction between the host Ni and the guest Sb due to the strong hybridization. X-ray photoelectron spectroscopy (XPS) analysis was thus employed to explore the electronic structures of the catalysts. The peaks positioned at binding energies of 852.3 and 855.9 eV in the Ni *2p* XPS spectrum of the Ni catalyst are attributed to Ni^0^ and Ni^2+^ (Fig. 4i), respectively. The presence of the Ni^2+^ species could be caused by the re-oxidation of the Ni catalyst during the ex-situ measurements^16,26^. Notably, the Ni *2p* XPS peaks of the NiSb catalyst shift to higher binding energies by 0.4 eV compared with those of the Ni catalyst. Meanwhile, the deconvoluted Sb *4d* XPS spectra exhibit four peaks corresponding to Sb^0^ and Sb^3+^ species (Fig. 4i), which shift to lower binding energy compared to those of the monometallic Sb^27^. The positive shift of Ni *2p* XPS peaks and simultaneously negative shift of Sb *4d* XPS peaks indicate electron transfer from Ni to Sb, in line with the higher electronegativity of the guest Sb (2.05) than that of the host Ni (1.91). The electronic properties and local environment of the NiSb catalyst were further revealed by X-ray absorption spectroscopy measurements. The normalized X-ray absorption near-edge structure (XANES) spectrum at the Ni K-edge of the NiSb catalyst shifts to the position of high photon energy with an increased intensity of white line peak compared to that of the Ni foil and the Ni catalyst (Fig. 4j), suggesting the decreased electron density of Ni atoms in the NiSb catalyst due to the electron transfer from Ni to the neighboring Sb^28–31^. The accumulated electron density on the guest Sb is favorable for binding the electron-deficient acetylene reactant but unfavorable for capturing the electron-rich ethylene product, which gives rise to the moderate σ-adsorption of acetylene and π-adsorption of ethylene on the trimer Ni_1_Sb_2_ site (Fig. 1b, c).

Furthermore, the Fourier transform of extended X-ray absorption fine structure (EXAFS) at the Ni K-edge of the NiSb catalyst exhibits lower intensity at the first nearest-neighbor coordination than that of the Ni catalyst (Fig. 4k), suggesting a decreased Ni-Ni coordination in the NiSb catalyst. More importantly, a remarkable peak associated with the NiSb coordination is observed at a longer distance, and the peak corresponding to the Ni-Ni coordination is absent in the EXAFS spectrum of the NiSb catalyst, evidencing the isolated Ni sites by Sb in the intermetallic phase. This is further confirmed by the EXAFS oscillation at the K space of the NiSb catalyst (Fig. 4l). The shorter periods and weaker amplitudes than those of the Ni foil and the referenced Ni catalyst indicate the longer coordination distance of NiSb and lower coordination environment in the NiSb catalyst^32–35^. Wavelet transforms (WT) analyses of the Ni EXAFS oscillations were further performed to gain more powerful evidence for strengthening the isolated Ni sites by Sb in the NiSb catalyst (Fig. 4m). The WT-EXAFS contour plots of the Ni foil and the Ni catalyst both show a maximum at around 8.2 Å^−1^ contributed by the Ni-Ni coordination. In contrast, the WT-EXAFS contour plot of the NiSb catalyst only exhibits a maximum at around 10.5 Å^−1^, which is assigned to the contribution of the Ni-Sb coordination. All the above structural characterizations clearly reveal the formation of NiSb intermetallic structure featured with the trimer Ni_1_Sb_2_ site in the synthesized NiSb catalyst.

### Catalytic performance of acetylene semi-hydrogenation

**Fig. 5:**
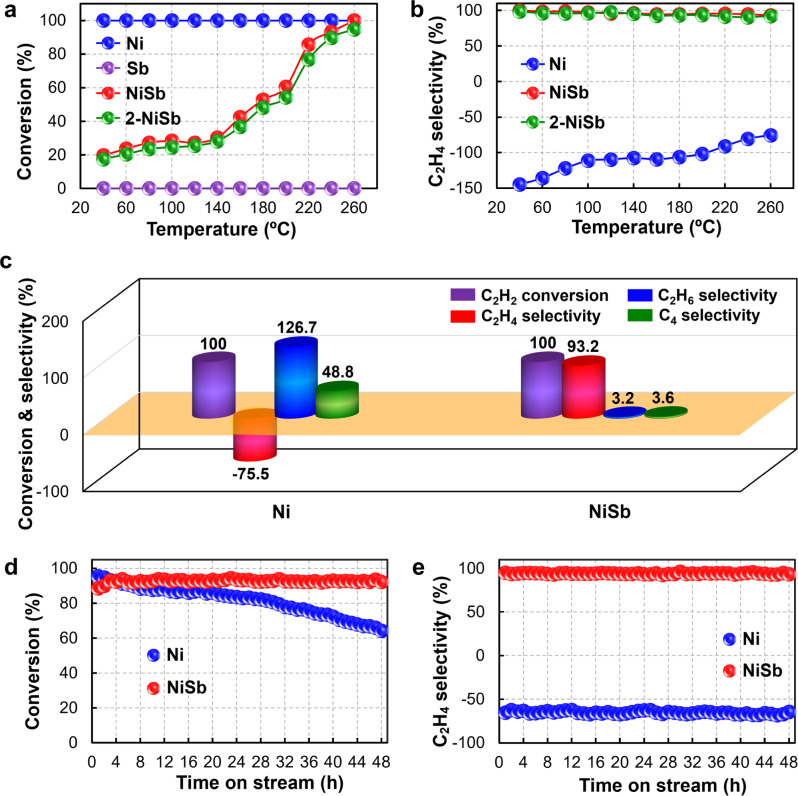
Catalytic performances for acetylene hydrogenation. **a** C_2_H_2_ conversion and **b** C_2_H_4_ selectivity as a function of reaction temperature; **c** Comparison for acetylene conversion and products selectivities of the Ni and NiSb catalysts; **d** Acetylene conversion and **e** ethylene selectivity with the time on stream over the Ni and NiSb catalysts.

The synthesized NiSb catalyst was employed for acetylene hydrogenation in the presence of ethylene together with the referred Ni catalyst. The conversion of acetylene over the NiSb catalyst gradually increases from 20 to 100% with the increasing reaction temperature from 40 to 260 °C, while the selectivity to ethylene maintains to be higher than 90% at such temperature range (Fig. 5a, b). In contrast, the conversion of acetylene over the monometallic Ni catalyst stays at around 100% at the whole temperature range, while that on the monometallic Sb catalyst is close to zero (Fig. 5a). In addition, the selectivity to ethylene increases with the increasing temperature on the Ni catalyst, probably due to the favored ethylene desorption^16^ and thus suppressed formation of ethane (Supplementary Fig. 30), but is still much lower than that of the NiSb catalyst (Fig. 5b). Moreover, the Ni catalyst exhibits remarkably higher selectivity to C_4_ components than the NiSb catalyst (Supplementary Fig. 31), indicating the promoted coupling process of C_2_ species on the Ni catalyst, which is a clue that the stability of the Ni catalyst may be inferior. It should be noted that the high conversion of acetylene on the Ni catalyst is owing to the facile formation of ethane and C_4_ components rather than the formation of targeted ethylene. The comparison for the calculated formation rate of the products demonstrates that the NiSb catalyst exhibits a remarkably enhanced formation rate of ethylene with clearly suppressed ones of ethane and C_4_ components than the Ni catalyst (Supplementary Fig. 38). Notably, the conversion of acetylene and the products selectivities over the 2-NiSb catalyst are close to those over the NiSb catalyst (Fig. 5a, b and Supplementary Figs. 30, 31), also confirming the formation of NiSb intermetallic phase in the 2-NiSb catalyst (Fig. 3a and Supplementary Fig. 17).

A more detailed comparison of the catalytic performances of the Ni and NiSb catalysts in Fig. 5c demonstrate that the NiSb catalyst exhibits an excellent ethylene selectivity up to 93.2% at the full conversion of acetylene, with the ethane selectivity and C_4_ selectivity low to 3.2 and 3.6%, respectively. However, the monometallic Ni catalyst shows significantly low ethylene selectivity (i.e., −75.5%) at 100% of acetylene conversion. This indicates that a large amount of ethylene in the feed gas was simultaneously hydrogenated to ethane, as evidenced by the high ethane selectivity of the Ni catalyst (Fig. 5c). In addition, the selectivity to C_4_ components of the Ni catalyst is clearly higher than that of the NiSb catalyst. Previous studies showed that the formation of C_4_ components in acetylene hydrogenation is mainly attributed to the C-C coupling of strongly adsorbed surface species such as acetylene and vinyl^36–39^. The higher selectivity to C_4_ components suggests a more facile coupling process on the Ni catalyst. Furthermore, the ethylene selectivity at the full conversion of acetylene on the NiSb intermetallic catalyst is clearly higher than that seen with the previously reported NiGa intermetallic catalyst featured with isolated Ni sites by neighboring Ga ones^16^. This comparison may indicate the unique electronic and geometric effects of the neighboring Sb sites in the NiSb intermetallics.

Arrhenius plots for acetylene conversion and product formation were further performed to explore the kinetics advantages of the NiSb catalyst (Supplementary Figs. 32–35). The determined apparent activation energy for acetylene conversion over the Ni (29.7 kJ/mol) is clearly lower than that on the NiSb catalyst (43.9 kJ/mol), which indicates that the activation of acetylene on the Ni catalyst is more facile. However, such easily activated acetylene is difficult to be converted to the targeted ethylene product, as revealed by the much higher apparent activation energy for the formation of ethylene on the Ni catalyst (Supplementary Fig. 33). Instead, the apparent activation energies for the formations of ethane and C_4_ components on the NiSb catalyst are obviously higher than those on the Ni catalyst (Supplementary Figs. 34,  35). These kinetics results demonstrate that the formations of ethane and C_4_ components are remarkably more inert on the NiSb catalyst. In contrast, the formation of targeted ethylene on the NiSb catalyst is more kinetically favorable than those of ethane and C_4_ components.

Considering that the C_4_ components are the precursors of green oil, the stability of the Ni catalyst would be inferior to that of the NiSb catalyst, which is addressed by the stability tests for the Ni and NiSb catalysts. As expected, the conversion of acetylene on the NiSb catalyst keeps at around 92.0% with 93.5% of ethylene selectivity (Fig. 5d, e) but negligible formation of ethane and C_4_ components through the 48 h stability testing (Supplementary Figs. 39–40), presenting an excellent catalytic stability. By contrast, the conversion of acetylene on the monometallic Ni catalyst decreases sharply from the initial 95.0% to around 64.0% after reaction for 48 h, while the selectivities to ethylene, ethane and C_4_ components are unchanged with the time on stream. These results distinctly demonstrate the deactivation of the Ni catalyst during the hydrogenation process, mainly due to the accumulated green oil on the surface.

**Fig. 6:**
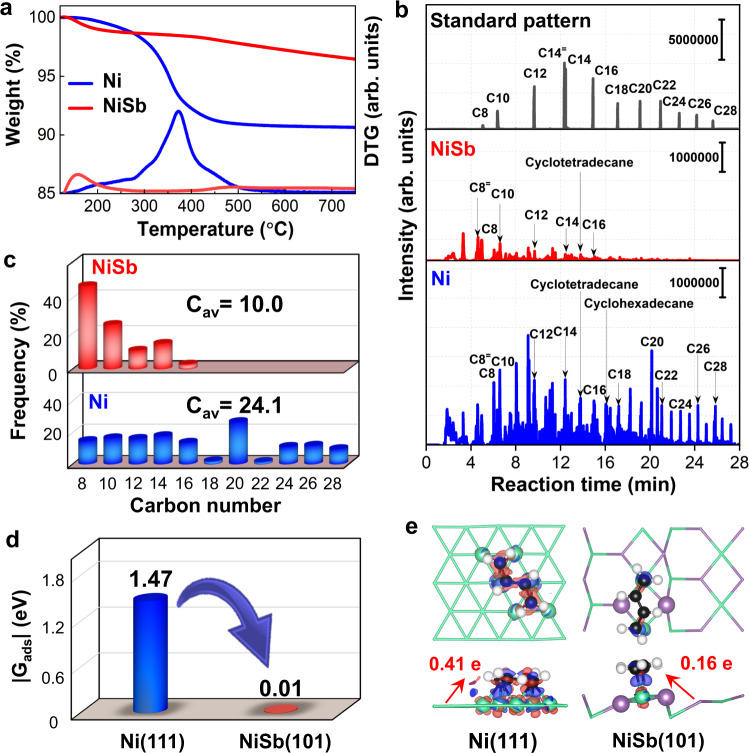
Coke analysis. **a** TG-DTG profiles of the spent Ni and NiSb catalysts. **b** Pyrolysis GC-MS profiles of green oil deposited on the Ni and NiSb catalysts where the standard pattern is taken from the refs. 41, 47 and **c** corresponding statistics for carbon numbers of hydrocarbons contained in green oil. **d** Adsorption-free energy of 1,3-butadiene over the Ni(111) and NiSb(101) surfaces. **e** Top and front views of the charge density distributions of 1,3-butadiene adsorbed on the Ni(111) and NiSb(101) surfaces. The red and blue isosurfaces represent the accumulation and depletion of the electron density, respectively, in which the isosurface value are both 0.005 e·Å^−3^. The red arrows indicate the direction of charge transfer between the surfaces and the 1,3-butadiene.

The thermogravimetric (TG) analyses were then performed for the spent Ni and NiSb catalysts to explore the possible deposition of green oil. The weight loss of the spent Ni catalyst with increasing the temperature is around 9.4 wt%, suggesting an obvious accumulation of green oil on the catalyst (Fig. 6a). Moreover, the DTG curve exhibits a sharp peak at ca. 375 °C with a peak shoulder at ca. 250 °C, which can be ascribed to the combustion of heavy and light hydrocarbons^16,40,41^, respectively. In contrast, the TG profile of the spent NiSb catalyst shows a slight weight loss at the range of 120–200 °C, mainly resulting from the vaporization of physiosorbed water. Notably, the peaks corresponding to the combustions of light and heavy hydrocarbons are hardly observed for the spent NiSb catalyst, indicating the negligible formation of green oil on the catalyst. Pyrolysis gas chromatography-mass spectrometer (GC-MS) experiments were further performed for the used Ni and NiSb catalysts to explore the compositions of formed green oil. Figure 6b shows the pyrolysis GC-MS profiles of these catalysts and that of a standard sample made up of various chain hydrocarbons. Clearly, the intensities of peaks observed with the spent Ni catalyst are much stronger than those seen with the used NiSb catalyst, indicating that more green oil was formed and accumulated on the Ni catalyst than the NiSb catalyst, which is in good accordance with the TG analyses. In addition, the green oil accumulated on the Ni catalyst contains more heavy hydrocarbons than that on the NiSb catalyst, as quantitatively confirmed by the statistical analysis for the carbon numbers of the chain hydrocarbons contained in the green oil (Fig. 6c). The averaged carbon number of the chain hydrocarbons comprised in the green oil on the used Ni catalyst is 24.1, clearly larger than that on the used NiSb catalyst. These unambiguously demonstrate the suppressed formation of green oil via the coupling process over the NiSb catalyst.

The origin of the restrained formation of green oil on the intermetallic NiSb surface is further traced by theoretical calculations. 1,3-butadiene as the precursor for the formation of green oil, which is formed via the coupling process, is revealed to adsorb on the Ni(111) surface via binding with six adjacent Ni atoms with an adsorption-free energy of −1.47 eV (Fig. 6d). In contrast, 1,3-butadiene binds weakly with two elongated Ni sites on the NiSb(101) surface with an adsorption-free energy of −0.01 eV. The weaker adsorption of 1,3-butadiene on the NiSb surface than that on the Ni surface is further evidenced by the less charge transfer between the molecule and the surface (Fig. 6e). These results elucidate that the introduction of Sb destabilizes the 1,3-butadiene molecule on the NiSb surface with isolated Ni sites, which is favorable for the desorption of 1,3-butadiene against its accumulation and polymerization to green oil. The performance tests strengthen the excellent selectivity of the trimer Ni_1_Sb_2_ site for acetylene semi-hydrogenation predicted by the theoretical calculations.

### Adsorption behaviors on trimer Ni_1_Sb_2_ site

**Fig. 7:**
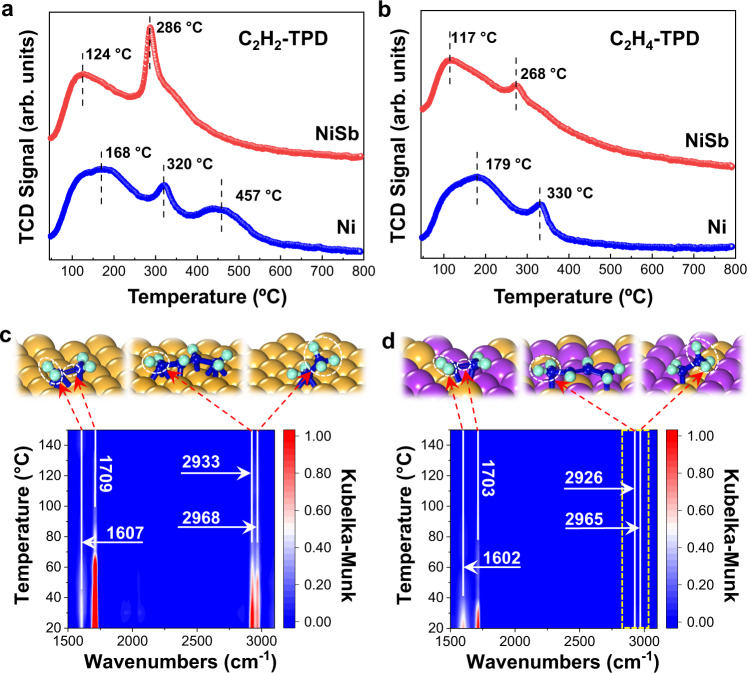
Adsorption/desorption and hydrogenation behaviors on Ni_1_Sb_2_. **a** C_2_H_2_-TPD and **b** C_2_H_4_-TPD profiles of the Ni and NiSb catalysts. In-situ DRIFTS spectra of the hydrogenation of pre-adsorbed acetylene on **c** Ni and **d** NiSb catalysts at elevated temperatures. The assignments of the characteristic peaks are schematically shown in the insets above the spectra.

Temperature-programmed desorption measurements were further carried out to explore the unique adsorption/desorption behaviors of C_2_H_2_ and C_2_H_4_ on the Ni and NiSb catalysts. As shown in Fig. 7a, the C_2_H_2_-TPD profile of the Ni catalyst presents three legible peaks centered at 168, 320, and 457 °C. According to previous studies, the peak at the temperature of 168 °C is attributed to the desorption of weakly π-adsorbed C_2_H_2_, and the peaks located at 320 and 457 °C are corresponded to the desorption of C_2_H_2_ species and/or the corresponding C_2_-fragments formed at the elevated temperature di-σ-bonded on bridge Ni sites and multi-σ-bonded on hollow Ni sites, respectively^42–44^. The C_2_H_2_-TPD profile of the NiSb catalyst shows two visible desorption peaks located at 124 and 286 °C corresponded to π-adsorbed and σ-adsorbed C_2_H_2_, respectively, which are lower than those of the peaks seen with the profile of the Ni catalyst. The peak corresponded on the species desorbed from the hollow site is hardly observed in the C_2_H_2_-TPD profile of the NiSb catalyst, suggesting the absence of multi-σ-bonded C_2_H_2_ on the hollow site, agreeing well with the results from theoretical calculations (Fig. 1b and Supplementary Tables 3–6). The C_2_H_4_-TPD profile of the Ni catalyst displays two peaks centered at 179 and 330 °C (Fig. 7b). According to previous studies^42–44^, the desorption peaks at the temperature below 300 °C are assigned to the continuous desorption of the weakly π-adsorbed C_2_H_4_ without decomposition, and those located at the temperature higher than 300 °C are assigned to the desorption of C_2_-fragments decomposed from the strongly σ-bonded C_2_H_4_. In contrast, the C_2_H_4_-TPD profile of the NiSb catalyst only demonstrates weakly π-adsorbed ethylene on the NiSb catalyst, which is also in line with the theoretical results.

The hydrogenation of pre-adsorbed acetylene on the catalysts were further traced by in situ diffuse reflectance infrared Fourier transform spectroscopy (DRIFTS) measurements at increasing temperature. Figure 7c, d and Supplementary Figs. 41, 42 show the spectra collected during the hydrogenation of pre-adsorbed acetylene over the Ni and NiSb catalysts at a temperature from 20 to 150 °C. The formation of ethylene on the Ni catalyst is evidenced by the characteristic peaks of the CH_2_ scission and the C=C stretching vibration^45^ at 1607 and 1709 cm^−1^, respectively. In addition, the peaks at 2933 and 2968 cm^−1^ corresponded to the -CH_2_ asymmetric stretching of long-chain hydrocarbons and -CH_3_ asymmetric stretching of alkanes^46^, respectively, indicate the facile coupling and over-hydrogenation processes on the Ni catalyst. These are in good accordance with observed higher selectivities to ethane and C_4_ components in Fig. 5. On the NiSb catalyst, the formation of ethylene is also confirmed by the two characterization peaks of the CH_2_ scission and the C=C stretching vibration at 1602 and 1703 cm^−1^. Notably, the characteristic peaks assigned to the alkanes and the long-chain hydrocarbons attenuate significantly on the NiSb catalyst and are almost hard to be observed from the spectra. These reveal the inhibited formations of ethane and the green oil precursor on the NiSb catalyst. Moreover, the intensities of the peaks corresponded to ethylene on the NiSb catalyst decrease with the increasing temperature more remarkably than those seen with the Ni catalyst, implying the weaker adsorption of ethylene on the NiSb catalyst. These results strengthen the excellent selectivity of the trimer Ni_1_Sb_2_ sites in the NiSb catalyst against the referred Ni catalyst.

In summary, we have employed the unique electronegative and *p*-block characteristics of guest Sb to regulate the host Ni to achieve a trimer Ni_1_Sb_2_ site in NiSb intermetallic with superior performance for acetylene semi-hydrogenation. Our theoretical results indicate that the trimer Ni_1_Sb_2_ site in the intermetallic *P6*_*3*_*/mmc* NiSb endows a moderate σ-adsorption mode for acetylene while a weak π-adsorption one for ethylene, implying boosted acetylene semi-hydrogenation. As predicted by the theoretical results, the NiSb catalyst featured with the trimer site fabricated by an in situ trapping strategy of molten Sb by Ni exhibits an excellent ethylene selectivity up to 93.2% with significantly low selectivities to ethane and C_4_ components at 100% of acetylene conversion, prevailing over the referred Ni catalyst. These findings exemplify the design and fabrication of atomically uniform active sites for fine-tuning configurations of key species to regulate the selectivity to the targeted product.

## Methods

### Synthesis of catalysts

Ni(NO_3_)_2_·6H_2_O (99.0%), Mg(NO_3_)_2_·6H_2_O (99.0%), and Al(NO_3_)_3_·9H_2_O (99.0%) were purchased from Sinopharm Chemical Reagent Limited Corporation. Na_2_CO_3_ (99.9%), NaOH (99.9%), and Sb powder (99.9%) were purchased from Aladdin. Ternary Ni/Mg/Al-LDHs was synthesized by a typical co-precipitation method. Typically, 0.04 mol of Na_2_CO_3_ was dissolved in 100 mL of ultra-pure water to make a basic solution denoted as A. Then, 0.01 mol of Ni(NO_3_)_2_·6H_2_O, 0.05 mol of Mg(NO_3_)_2_·6H_2_O, and 0.02 mol of Al(NO_3_)_3_·9H_2_O were dissolved in 100 mL of ultra-pure water to obtain a mixed metal salt solution denoted as B. 0.25 mol of NaOH was subsequently dissolved in 250 mL of ultra-pure water as another solution denoted as C. Afterward, solution B and C were added dropwise into the solution A under vigorous stirring at 65 °C, during which the pH of the solution was maintained at 10. Thereafter, the mixture was aged at 65 °C for 16 h under vigorous stirring and then was filtered and completely washed with ultra-pure water until the pH is around 7. After being dried at 110 °C for 12 h under static air, the obtained solid sample was grinded and then soaked in a solution of Na_2_CO_3_ for 12 h. The mixture was filtered and washed with a great amount of ultra-pure water to remove the basic residues. Finally, the ternary Ni/Mg/Al-LDHs was obtained after drying at 110 °C for 12 h.

The Ni/Mg/Al-LDHs was reduced at 900 °C for 4 h to obtain the monometallic Ni catalyst. The precursor of the NiSb intermetallic catalyst was gained by mixing the Ni/Mg/Al-LDHs and Sb powder with Sb/Ni molar ratios of 1.0 and 2.0 by thoroughly grinding. The above mixture was reduced at 900 °C for 4 h to obtain NiSb intermetallic catalysts, which is denoted as NiSb and 2-NiSb, respectively. The binary Mg/Al-LDHs was also synthesized with a procedure similar to that of Ni/Mg/Al-LDHs without the addition of Ni(NO_3_)_2_·6H_2_O. The Mg/Al-LDHs was mixed with Sb powder by completely grinding and then reduced at 900 °C for 4 h to obtain the monometallic Sb catalyst. More details for the synthesis of single-atom Ni catalyst are shown in the Supplementary Materials.

### Characterizations of the materials

X-ray diffraction (XRD) characterization was conducted on a D8 ADVANCE diffractometer with Cu Kα radiation at 40 kV and 40 mA. The actual loading of Ni, Sb, Mg and Al in the Ni, Sb, 2-NiSb and intermetallic NiSb catalyst was measured by inductively coupled plasma atomic emission spectrometer (ICP-AES) on Varian 710-ES. N_2_ adsorption and desorption isotherms were performed on an ASAP-2020 instrument using the Brunauer–Emmett–Teller (BET) method to examine the textural properties of samples. The high-resolution transmission electron microscopy (HRTEM) images and high-angle annular dark-field scanning transmission electron microscopy (HAADF-STEM) analysis were carried out on a JEOL JEM-2010 F transmission electron microscope. Aberration-corrected HAADF-STEM (AC-HAADF-STEM) images were obtained from a Hitachi HF5000 scanning transmission electron microscope with a Cs corrector working at 200 kV. X-ray photoelectron spectroscopy (XPS) tests were performed on a Thermo Scientific ESCALAB 250xi system with radiation of Al Ka. The corresponding binding energies of samples were calibrated to the C 1 s peak at 284.6 eV. XAFS measurements at the Ni K-edge (8333 eV) were tested at the BL11B XAFS beamline of Shanghai Synchrotron Radiation Facility (SSRF). Analysis of green oil composition was performed by pyrolysis GC-MS (Agilent 7890 A GC/5975 C MSD) equipped with an HP-5MS column. The sample of spent catalyst was heated at 600 °C to pyrolyze the deposited green oil on the catalysts^41,47^. The thermogravimetric analysis tests were carried out with a PerkinElmer Pyris 1 by increasing the temperature from room temperature to 800 °C with a rate of 10 °C/min.

C_2_H_2_-TPD measurements for the catalysts were carried out on a Micrometrics Autochem II 2920 chemisorption system equipped with a thermal conductivity detector (TCD). About 100 mg of the sample reduced at 900 °C for 4 h was pretreated with 5 vol% H_2_/Ar with a flow rate of 45 mL/min at 800 °C for 3 h. Afterwards, the sample was purged with pure Ar and then cooled down to 45 °C. 4.0 vol% C_2_H_2_/N_2_ at a flow rate of 30 mL/min was introduced to the sample for 1 h to ensure a saturated adsorption, and then pure Ar was flowed to remove gas-phase C_2_H_2_. The C_2_H_2_-TPD profiles were subsequently obtained by increasing the temperature from 50 to 800 °C at a rate of 10 °C/min. For C_2_H_4_-TPD, the sample was also pretreated in the same way as that for C_2_H_2_-TPD. Then, the sample was exposed to 20.0 vol% C_2_H_4_/N_2_ at a flow rate of 30 mL/min for 1 h followed by being flushed with pure Ar to remove gas-phase C_2_H_4_. The C_2_H_4_-TPD profiles were also collected by increasing the temperature from 50 to 800 °C at a rate of 10 °C/min.

In-situ DRIFTS spectra for acetylene hydrogenation were collected in an in situ diffuse reflection cell (Harrick Praying Mantis) placed in PerkinElmer Spectrum 100 FTIR spectrometer. Typically, about 50 mg of catalyst powders pre-reduced at 900 °C for 4 h were flowed with 20.0 vol% H_2_/N_2_ with a flow rate of 30 mL/min at 500 °C for 2 h, and then cooled down to 20 °C under pure Ar. Thereafter, 4.0 vol% C_2_H_2_/N_2_ was introduced into the reflection cell with a flow rate of 20 mL/min for 1 h to ensure the saturated adsorption of acetylene on the sample. Finally, 5.0 vol% H_2_/N_2_ with a flow rate of 20 mL/min was flowed to the cell for hydrogenation of the pre-adsorbed acetylene, during which the temperature was increased gradually to 150 °C. The spectra collected at different temperature were subtracted from background spectra at the corresponding temperature.

### Acetylene hydrogenation testing

The catalytic performance tests were performed in a tubular stainless reactor. For the Ni, Sb, NiSb and 2-NiSb catalysts, about 300 mg of catalyst sample was loaded in the center of the stainless-steel tube, reduced in 20 vol% H_2_/N_2_ using a flow rate of 60 mL/min at 800 °C for 3 h, and cooled down to initial reaction temperature in N_2_ with a flow rate of 30 mL/min. Thereafter, the feed gas composed of 0.5 vol% acetylene, 2.5 vol% hydrogen, 30.0 vol% ethylene and balanced N_2_ was introduced into the reactor at a flow rate of 30 mL/min. The composition of the reactants and products were analyzed online by a INFICON 3000 Micro gas chromatograph equipped with a TCD detector. The acetylene conversion and ethylene selectivity were calculated as follows:

1 $${{{{{{\mathrm{C}}}}}}}_{2}{{{{{{\mathrm{H}}}}}}}_{2}\,{{{{{{\mathrm{conversion}}}}}}}=\frac{{{{{{{\mathrm{C}}}}}}}_{2}{{{{{{\mathrm{H}}}}}}}_{2}\left({{{{{{\mathrm{inlet}}}}}}}\right)-{{{{{{\mathrm{C}}}}}}}_{2}{{{{{{\mathrm{H}}}}}}}_{2}\left({{{{{{\mathrm{outlet}}}}}}}\right)}{{{{{{{\mathrm{C}}}}}}}_{2}{{{{{{\mathrm{H}}}}}}}_{2}\left({{{{{{\mathrm{inlet}}}}}}}\right)}\times 100\%$$

2 $$S\left({{{{{{\mathrm{C}}}}}}}_{2}{{{{{{\mathrm{H}}}}}}}_{4}\right)=\left\{1-\frac{{{{{{{\mathrm{C}}}}}}}_{2}{{{{{{\mathrm{H}}}}}}}_{6}\left({{{{{{\mathrm{outlet}}}}}}}\right){-}{{{{{{\mathrm{C}}}}}}}_{2}{{{{{{\mathrm{H}}}}}}}_{6}\left({{{{{{\mathrm{inlet}}}}}}}\right)}{{{{{{{\mathrm{C}}}}}}}_{2}{{{{{{\mathrm{H}}}}}}}_{2}\left({{{{{{\mathrm{inlet}}}}}}}\right){-}{{{{{{\mathrm{C}}}}}}}_{2}{{{{{{\mathrm{H}}}}}}}_{2}\left({{{{{{\mathrm{outlet}}}}}}}\right)}-\frac{2\times \left[{{{{{{\mathrm{C}}}}}}}_{4}\left({{{{{{\mathrm{outlet}}}}}}}\right){-}{{{{{{\mathrm{C}}}}}}}_{4}\left({{{{{{\mathrm{inlet}}}}}}}\right)\right]}{{{{{{{\mathrm{C}}}}}}}_{2}{{{{{{\mathrm{H}}}}}}}_{2}\left({{{{{{\mathrm{inlet}}}}}}}\right){-}{{{{{{\mathrm{C}}}}}}}_{2}{{{{{{\mathrm{H}}}}}}}_{2}\left({{{{{{\mathrm{outlet}}}}}}}\right)}\right\}\times 100\%$$

3 $$S\left({{{{{{\mathrm{C}}}}}}}_{2}{{{{{{\mathrm{H}}}}}}}_{6}\right)=\frac{{{{{{{\mathrm{C}}}}}}}_{2}{{{{{{\mathrm{H}}}}}}}_{6}\left({{{{{{\mathrm{outlet}}}}}}}\right){-}{{{{{{\mathrm{C}}}}}}}_{2}{{{{{{\mathrm{H}}}}}}}_{6}\left({{{{{{\mathrm{inlet}}}}}}}\right)}{{{{{{{\mathrm{C}}}}}}}_{2}{{{{{{\mathrm{H}}}}}}}_{2}\left({{{{{{\mathrm{inlet}}}}}}}\right){-}{{{{{{\mathrm{C}}}}}}}_{2}{{{{{{\mathrm{H}}}}}}}_{2}\left({{{{{{\mathrm{outlet}}}}}}}\right)}\times 100\%$$

4 $$S\left({{{{{{\mathrm{C}}}}}}}_{4}\right)=\frac{2\times \left[{{{{{{\mathrm{C}}}}}}}_{4}\left({{{{{{\mathrm{outlet}}}}}}}\right){-}{{{{{{\mathrm{C}}}}}}}_{4}\left({{{{{{\mathrm{inlet}}}}}}}\right)\right]}{{{{{{{\mathrm{C}}}}}}}_{2}{{{{{{\mathrm{H}}}}}}}_{2}\left({{{{{{\mathrm{inlet}}}}}}}\right){-}{{{{{{\mathrm{C}}}}}}}_{2}{{{{{{\mathrm{H}}}}}}}_{2}\left({{{{{{\mathrm{outlet}}}}}}}\right)}\times 100\%$$

As shown in Supplementary Table 12, at around 10% of acetylene conversion, the carbon balances were calculated to be 99.47 and 99.98% for the Ni and NiSb catalysts, respectively. At around 90% of acetylene conversion, the carbon balances were calculated to be 97.81 and 99.93% for the Ni and NiSb catalysts, respectively. The lower carbon balance for the Ni catalyst suggests more carbonaceous formed on the catalyst, which is confirmed by the TG analysis for the used Ni catalyst.

### DFT calculations

All DFT calculations were carried out with the Vienna Ab initio Simulation Package (VASP)^48–50^ with plane wave basis sets and projected-augmented wave (PAW) pseudopotentials^51^. The generalized gradient approximation (GGA) proposed by Perdew–Burke–Ernzerhof (PBE)^52^ was employed for the exchange-correlation functionals. The initial structures of bulk Ni and NiSb alloy taken from the Materials Project^53^ were optimized. The most thermodynamically stable Ni(111) and NiSb(101) surface were studied for DFT calculations. Ni(111) surface was modeled with four layers in *p*(3 × 3) supercells. NiSb(101) and NiSb(102) surfaces were modeled with four layers in *p*(2 × 3) supercells. The top two layers were relaxed, and the others were fixed at the bulk lattice positions. The NiSb(100) surface was modeled with two layers in *p*(3 × 3) supercells. The top layer was relaxed, and the other was fixed at the bulk lattice positions. A vacuum layer of 20 Å was set between the periodically repeated slabs to avoid interactions from adjacent cells. The transition states were obtained from adopting dimer method^54^ and confirmed to have only one imaginary frequency through the vibrational frequency analysis. Bader analysis^55^ was carried out to calculate atomic electronic charges for verifying electronic interaction between adsorbed species and the metal surfaces. More details including the calculations for the adsorption energy and reaction barrier are shown in the Supplementary Materials.

## Supplementary information

Supplementary Information

## Data Availability

The authors declare that all the important data to support the findings in this paper can be found in the main text or Supplementary information. Extra data were available from the corresponding author upon reasonable request.

## References

[1] Wang A, Li J, Zhang T (2018). Heterogeneous single-atom catalysis. Nat. Rev. Chem..

[2] Qin R, Liu K, Wu Q, Zheng N (2020). Surface coordination chemistry of atomically dispersed metal catalysts. Chem. Rev..

[3] Ji S (2020). Chemical synthesis of single atomic site catalysts. Chem. Rev..

[4] Zhang L, Zhou M, Wang A, Zhang T (2020). Selective hydrogenation over supported metal catalysts: from nanoparticles to single atoms. Chem. Rev..

[5] Feng Q (2019). Mesoporous nitrogen-doped carbon-nanosphere-supported isolated single-atom Pd catalyst for highly efficient semihydrogenation of acetylene. Adv. Mater..

[6] Huang F (2018). Atomically dispersed Pd on nanodiamond/graphene hybrid for selective hydrogenation of acetylene. J. Am. Chem. Soc..

[7] Huang F (2019). Anchoring Cu_1_ species over nanodiamond-graphene for semi-hydrogenation of acetylene. Nat. Commun..

[8] Deng, X. et al. Homogeneous-like alkyne selective hydrogenation catalyzed by cationic nickel confined in zeolite. *CCS Chem*. **4**, 1101–1114 (2021).

[9] Chai Y (2019). Acetylene-selective hydrogenation catalyzed by cationic nickel confined in zeolite. J. Am. Chem. Soc..

[10] Ji S (2019). Atomically dispersed ruthenium species inside metal-organic frameworks: combining the high activity of atomic sites and the molecular sieving effect of MOFs. Angew. Chem. Int. Ed..

[11] Gu J (2021). Synergizing metal-support interactions and spatial confinement boosts dynamics of atomic nickel for hydrogenations. Nat. Nanotechnol..

[12] Feng Q (2017). Isolated single-atom Pd sites in intermetallic nanostructures: high catalytic selectivity for semihydrogenation of alkynes. J. Am. Chem. Soc..

[13] Wei S (2018). Direct observation of noble metal nanoparticles transforming to thermally stable single atoms. Nat. Nanotechnol..

[14] Zhou H (2016). PdZn intermetallic nanostructure with Pd-Zn-Pd ensembles for highly active and chemoselective semi-hydrogenation of acetylene. ACS Catal..

[15] Spanjers CS (2014). Zinc inclusion to heterogeneous nickel catalysts reduces oligomerization during the semi-hydrogenation of acetylene. J. Catal..

[16] Cao Y (2020). Adsorption site regulation to guide atomic design of Ni-Ga catalysts for acetylene semi-hydrogenation. Angew. Chem. Int. Ed..

[17] Ge, X. et al. Enhanced acetylene semi-hydrogenation on subsurface carbon tailored Ni-Ga intermetallic catalyst. *J. Mat. Chem. A*10.1039/d2ta02216h (2022).

[18] Katsuyama S, Watanabe M, Kuroki M, Maehata T, Ito M (2003). Effect of NiSb on the thermoelectric properties of skutterudite CoSb_3_. J. Appl. Phys..

[19] Kumari L, Li W, Huang JY, Provencio PP (2010). Nanosize transition metal antimonides, NiSb and FeSb_2_: solvothermal synthesis and characterization. J. Phys. Chem. C..

[20] Li C, Hu J, Peng Q, Wang X (2008). Synthesis and characterization of nanocrystalline NiSb and NiSb_2_ at low temperature. Mater. Chem. Phys..

[21] Xie J (2007). Low temperature solvothermal synthesis of nanosized NiSb as a Li-ion battery anode material. J. Alloy. Compd..

[22] Yang B (2013). Evidence to challenge the universality of the Horiuti-Polanyi mechanism for hydrogenation in heterogeneous catalysis: origin and trend of the preference of a non-Horiuti-Polanyi mechanism. J. Am. Chem. Soc..

[23] Zhao Z-J (2019). Competition of C-C bond formation and C-H bond formation for acetylene hydrogenation on transition metals: a density functional theory study. AlChE J..

[24] Rao DM (2018). The reaction mechanism and selectivity of acetylene hydrogenation over Ni-Ga intermetallic compound catalysts: a density functional theory study. Dalton Trans..

[25] Kift RL, Prior TJ (2010). Reductive synthesis of metal antimonides. J. Alloy. Compd..

[26] Liu Y (2018). Layered double hydroxide-derived Ni-Cu nanoalloy catalysts for semi-hydrogenation of alkynes: Improvement of selectivity and anti-coking ability via alloying of Ni and Cu. J. Catal..

[27] Fan G (2021). Nanoporous NiSb to enhance nitrogen electroreduction via tailoring competitive adsorption sites. Adv. Mater..

[28] Liu W (2021). Atomically-ordered active sites in NiMo intermetallic compound toward low-pressure hydrodeoxygenation of furfural. Appl. Catal. B.

[29] Yang Y (2018). Selective hydrogenation of cinnamaldehyde over Co-based intermetallic compounds derived from layered double hydroxides. ACS Catal..

[30] Yu J (2020). NiBi intermetallic compounds catalyst toward selective hydrogenation of unsaturated aldehydes. Appl. Catal. B.

[31] Han A (2019). Isolating contiguous Pt atoms and forming Pt-Zn intermetallic nanoparticles to regulate selectivity in 4-nitrophenylacetylene hydrogenation. Nat. Commun..

[32] Penner-Hahn JE (1999). X-ray absorption spectroscopy in coordination chemistry. Coord. Chem. Rev..

[33] Rehr JJ, Albers RC (2000). Theoretical approaches to x-ray absorption fine structure. Rev. Mod. Phys..

[34] Frenkel AI, Yevick A, Cooper C, Vasic R (2011). Modeling the structure and composition of nanoparticles by extended X-ray absorption fine-structure spectroscopy. Annu. Rev. Anal. Chem..

[35] Frenkel AI (2012). Applications of extended X-ray absorption fine-structure spectroscopy to studies of bimetallic nanoparticle catalysts. Chem. Soc. Rev..

[36] Yang B, Burch R, Hardacre C, Hu P, Hughes P (2014). Mechanistic study of 1,3-butadiene formation in acetylene hydrogenation over the Pd-based catalysts using density functional calculations. J. Phys. Chem. C..

[37] Vignola E (2018). Evaluating the risk of C-C bond formation during selective hydrogenation of acetylene on palladium. ACS Catal..

[38] Zhao ZJ (2018). Competition of C-C bond formation and C-H bond formation For acetylene hydrogenation on transition metals: a density functional theory study. AIChE J..

[39] Wang Y, Liu B, Lan X, Wang T (2021). Subsurface carbon as a selectivity promotor to enhance catalytic performance in acetylene semihydrogenation. ACS Catal..

[40] Kim W (2004). Deactivation behavior of a TiO_2_-added Pd catalyst in acetylene hydrogenation. J. Catal..

[41] Cao Y, Sui Z, Zhu Y, Zhou X, Chen D (2017). Selective hydrogenation of acetylene over Pd-In/Al_2_O_3_ catalyst: promotional effect of indium and composition-dependent performance. ACS Catal..

[42] Cao Y (2021). Structural and kinetics understanding of support effects in Pd-catalyzed semi-hydrogenation of acetylene. Engineering.

[43] Guan Q (2020). Selective hydrogenation of acetylene to ethylene over the surface of sub-2 nm Pd nanoparticles in *Miscanthus sinensis*-derived microporous carbon tubes. ACS Sustain. Chem. Eng..

[44] Chen M, Goodman DW (2008). Promotional effects of Au in Pd-Au catalysts for vinyl acetate synthesis. Chin. J. Catal..

[45] Moon J (2020). Discriminating the role of surface hydride and hydroxyl for acetylene semihydrogenation over ceria through in situ neutron and infrared spectroscopy. ACS Catal..

[46] Hu N, Yang C, He L, Guan Q, Miao R (2019). Ni-Cu/Al_2_O_3_ catalysts for the selective hydrogenation of acetylene: a study on catalytic performance and reaction mechanism. N. J. Chem..

[47] Zhang J (2016). Composition of the green oil in hydrogenation of acetylene over a commercial Pd-Ag/Al_2_O_3_ catalyst. Chem. Eng. Technol..

[48] Kresse G, Furthmüller J (1996). Efficiency of ab-initio total energy calculations for metals and semiconductors using a plane-wave basis set. Comput. Mater. Sci..

[49] Kresse G, Furthmüller J (1996). Efficient iterative schemes for ab initio total-energy calculations using a plane-wave basis set. Phys. Rev. B.

[50] Kresse G, Joubert D (1999). From ultrasoft pseudopotentials to the projector augmented-wave method. Phys. Rev. B.

[51] Blochl PE (1994). Projector augmented-wave method. Phys. Rev. B Condens. Matter Mater. Phys..

[52] Perdew JP, Burke K, Ernzerhof M (1996). Generalized gradient approximation made simple. Phys. Rev. Lett..

[53] Jain, A. et al. Commentary: The Materials Project: a materials genome approach to accelerating materials innovation. *APL Mater*. **1**, 011002 (2013).

[54] Henkelman G, JóNsson H, Henkelman G, Jónsson H (1999). A dimer method for finding saddle points on high dimensional potential surfaces using only first derivatives. J. Chem. Phys..

[55] Tang W, Sanville E, Henkelman G (2009). A grid-based Bader analysis algorithm without lattice bias. J. Phys. Condens. Matter.

