# Supplementary material for: Mechanism driven design of trimer Ni1Sb2 site delivering superior hydrogenation selectivity to ethylene
Source: Nat Commun. 2022 Sep 21;13:5534. doi: 10.1038/s41467-022-33250-8 (PMC9492709; doi:10.1038/s41467-022-33250-8)
Supplement: Supplementary file 1 — Supplementary Information [file 41467_2022_33250_MOESM1_ESM.pdf]

Supplementary materials

Mechanism Driven Design of Trimer Ni_1Sb_2 Site Delivering Superior Hydrogenation Selectivity to Ethylene

Xiaohu Ge[1], Mingying Dou[1], Yueqiang Cao[1,*], Xi Liu[2,*], Qiang Yuwen[1], Jing Zhang[1], Gang Qian[1], Xueqing Gong[3], Xinggui Zhou[1], Liwei Chen[2], Weikang Yuan[1], Xuezhi Duan[1,*]

[1] *State Key Laboratory of Chemical Engineering, East China University of Science and Technology, 130 Meilong Road, Shanghai 200237, China*

[2] *School of Chemistry and Chemical Engineering, In-situ Center for Physical Sciences, Frontiers Science Center for Transformative Molecules, Shanghai Jiao Tong University, Shanghai 200240, China*

[3] *Key Laboratory for Advanced Materials, Centre for Computational Chemistry and Research Institute of Industrial Catalysis, East China University of Science and Technology, 130 Meilong Road, Shanghai 200237, China*

[*] *Corresponding Authors:*

yqcao@ecust.edu.cn; liuxi@sjtu.edu.cn; xzduan@ecust.edu.cn

Supplementary Methods

All DFT calculations were carried out with the Vienna Ab initio Simulation Package (VASP) with plane wave basis sets and projected-augmented wave (PAW) pseudopotentials. The generalized gradient approximation (GGA) proposed by Perdew-Burke-Ernzerhof (PBE) was employed for the exchange-correlation functionals. The kinetic cut-off energy of 450 eV was set for all calculations, and spin polarization was considered. The DFT-D3 method of Grimme[1] was added to check the effect of van der Waals interaction on reaction energetics, and 3×3×1 Monkhorst-Pack k-point mesh was employed to sample the surface Brillouin zone[2]. A threshold of 10^{-5} eV was adopted for the convergence criterion of the electronic structure. The forces on the geometric optimization should be less than 0.05 eV/Å.

The initial structures of bulk Ni and NiSb alloy taken from the Materials Project were optimized. The most thermodynamically stable Ni(111) and NiSb(101) surface were studied for DFT calculations. Ni(111) surface was modeled with four layers in p(3×3) supercells. NiSb(101) and NiSb(102) surfaces were modeled with four layers in p(2×3) supercells. The top two layers were relaxed, and the others were fixed at the bulk lattice positions. The NiSb(100) surface was modeled with two layers in p(3×3) supercells. The top layer was relaxed, and the other was fixed at the bulk lattice positions. A vacuum layer of 20 Å was set between the periodically repeated slabs to avoid interactions from adjacent cells. The adsorption energy and reaction barrier were calculated by using the equation $E_{ads} = E_{adsorbate/surface} - E_{surface} - E_{adsorbate}$ and $E_a = E_{TS} - E_{IS}$, where $E_{adsorbate/surface}$, $E_{surface}$, $E_{adsorbate}$, E_{IS} and E_{TS} are the total energy of the surface covered with the adsorbate, the clean surface slab, a gaseous adsorbate molecule, the energies of the corresponding initial state and transition state, respectively. The adsorption free energy of the adsorbate was calculated as: $G_{ads} = G_{adsorbate/surface} - G_{surface} - G_{adsorbate}$, where $G_{adsorbate/surface}$, $G_{surface}$, $G_{adsorbate}$ is the total Gibbs free energy of the surface with the attached adsorbate, the clean surface slab, and a gas phase adsorbate molecule at 383.15 K and 1 bar, respectively.The Gibbs free energy barrier is calculated with the following equation $G_a = G_{TS} - G_{IS},$ where G_{IS} and G_{TS} are the preceding lowest energy intermediate (IS) and transition state (TS), respectively. The transition states were obtained from adopting dimer method and confirmed to have only one imaginary frequency through the vibrational frequency analysis.

Bader analysis was carried out to calculate atomic electronic charges for verifying electronic interaction between adsorbed species and the metal surfaces. For the electron transfer between these

metal surfaces and the adsorbed species, the charge density difference isosurface (Δρ) is calculated as *Δρ=Δρ(species/surface)-Δρ(surface)-Δρ(species)*, where *Δρ(species/surface)*, *Δρ(surface)* and *Δρ(species)* are the charge density of the species/surface system, the charge density of the relaxed surface and the charge density of adsorbed species in their optimized configuration, respectively.

Preparation and performance tests of the Ni-SA catalyst

The Al_2O_3 was employed as the substrate for synthesizing the Ni-SA catalyst by atomic layer deposition (ALD) method. ALD process was carried out using a viscous flow reactor (D100-4882, China) with nickelocene as the precursor for Ni. Ultrahigh purity N_2 (99.999%) was used as a carrier gas at a flow rate of 50 $mL{\cdot}min^{-1}$. The nickelocene precursor container was heated to 70 °C to reach a sufficient vapor pressure. The chamber was heated to 250 °C, and the inlet manifold was held at 150 °C to avoid the possible precursor condensation. The timing sequence was 12, 25, 12 and 25 s for nickelocene exposure, N_2 purge, O_3 exposure, and N_2 purge, respectively. Three ALD cycles were conducted here to synthesize Ni-SA catalyst. About 200 mg of Ni-SA catalyst was reduced in 20 vol% H_2/N_2 using a flow rate of 60 mL/min at 300 °C for 2h, and cooled down to initial reaction temperature in N_2 with a flow rate of 30 mL/min. Thereafter, the feed gas composed of 0.5 vol% acetylene, 2.5 vol% hydrogen, 30.0 vol% ethylene and balanced N_2 was introduced into the reactor at a flow rate of 20 mL/min. The composition of the reactants and products were analyzed online by a INFICON 3000 Micro gas chromatograph equipped with a TCD detector.

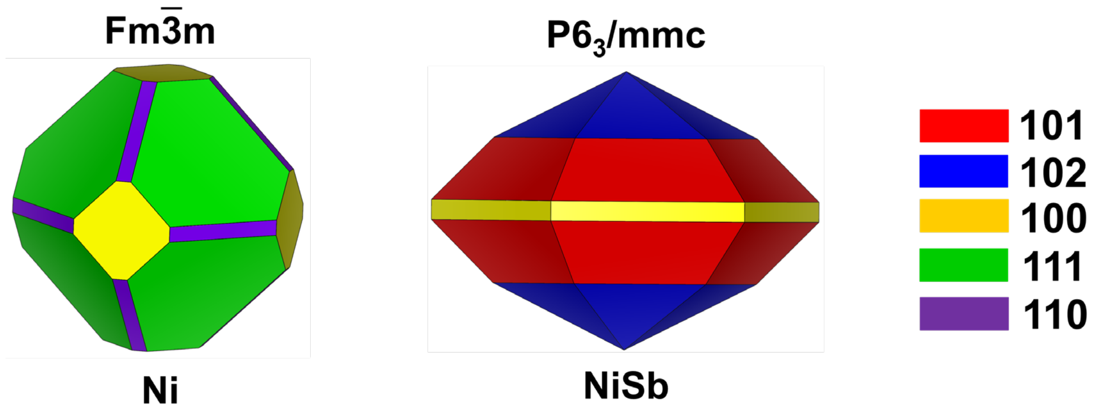

Supplementary Fig. 1. Wulff constructions for Ni and NiSb. Wulff crystals for Ni and NiSb crystallites, where the crystal facets denoted by Miller indices are shown by different colors.

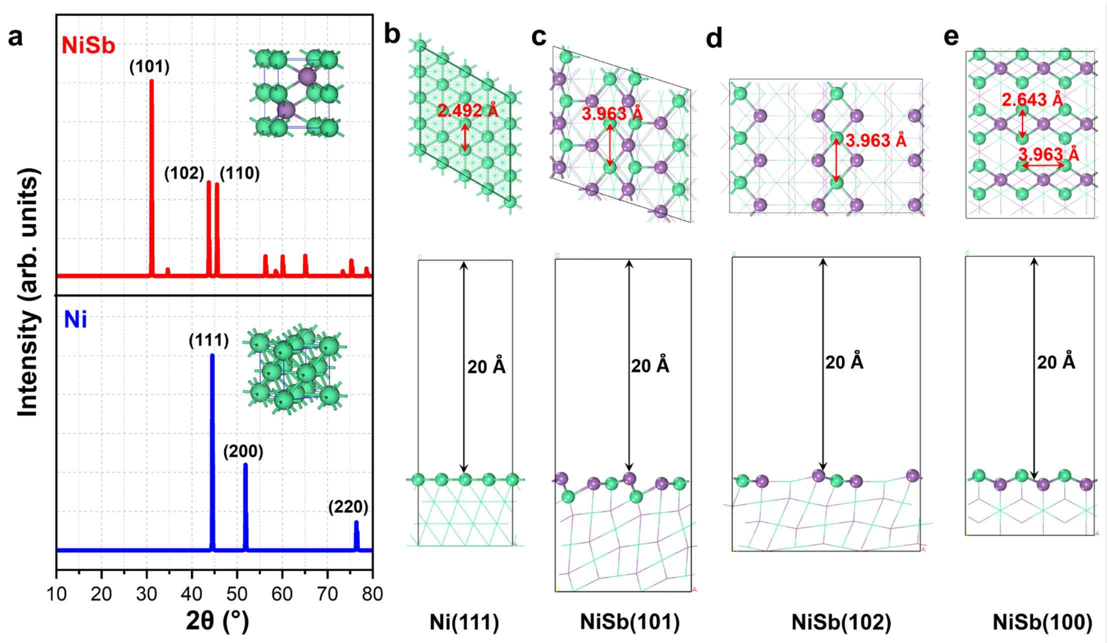

Supplementary Fig. 2. Crystal models and structures of Ni and NiSb. (a) Simulated XRD patterns of face-centered cubic Ni and hexagonal NiSb intermetallics with the corresponding crystal structures shown in the insets. The green and purple balls represent the Ni and Sb atoms, respectively. Schematical illustrations of (b) Ni(111), (c) NiSb(101), (d) NiSb(102) and (e) NiSb(100) surfaces.

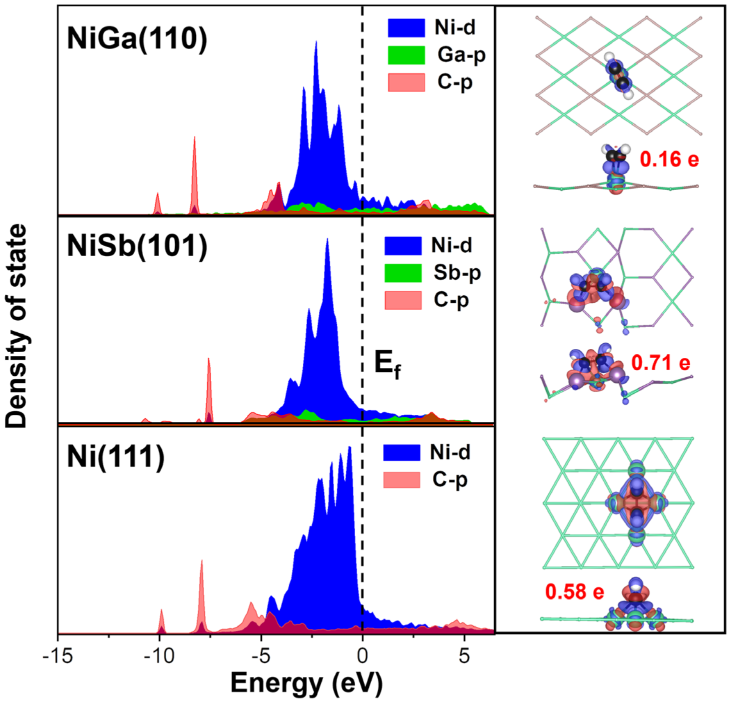

Supplementary Fig. 3. Electronic structures. Density of states and Bader charge analyses for acetylene adsorbed on the Ni(111), NiSb(101) and NiGa(110) surfaces. The Bader charge analysis for acetylene adsorbed on the NiGa(110) surface was taken from our previous work[3].

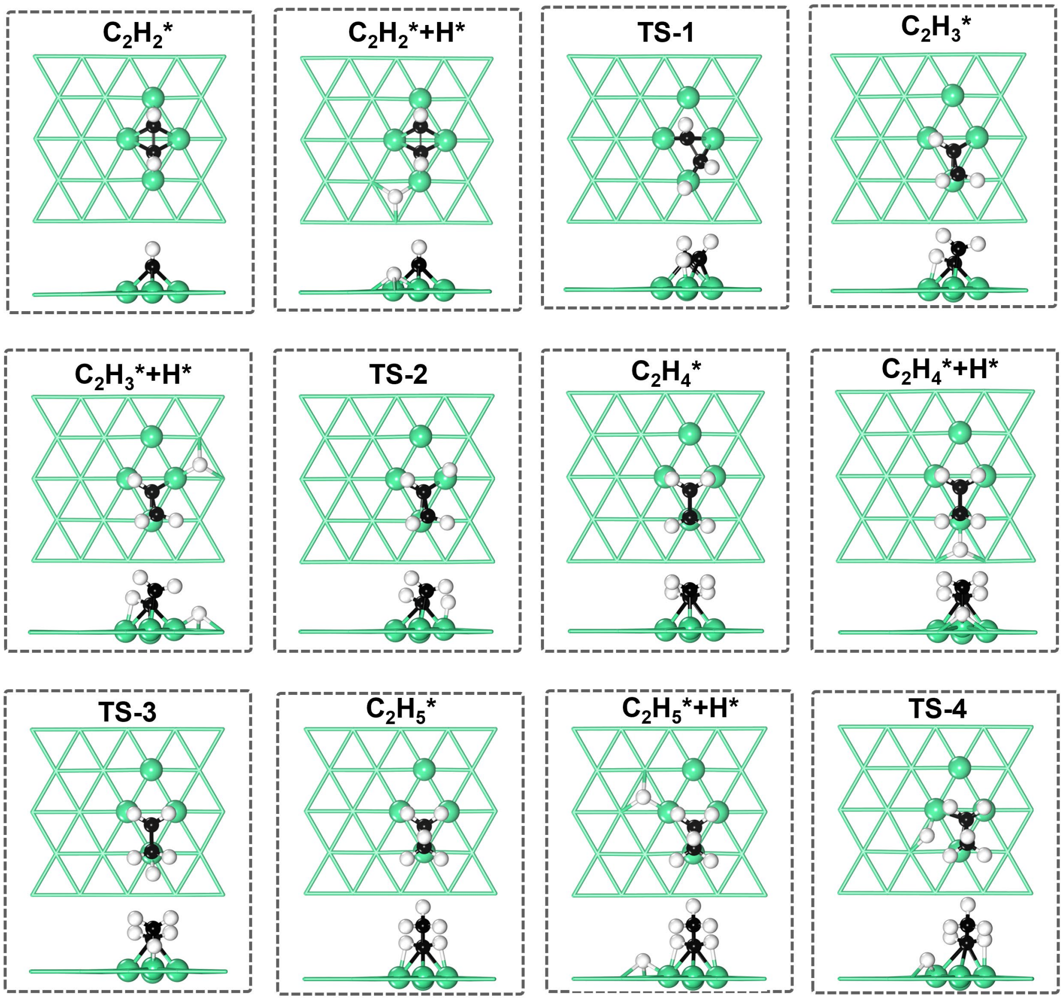

Supplementary Fig. 4. Intermediates on Ni(111). Configurations of the intermediates involved in the elementary steps of acetylene hydrogenation on the Ni(111) surface.

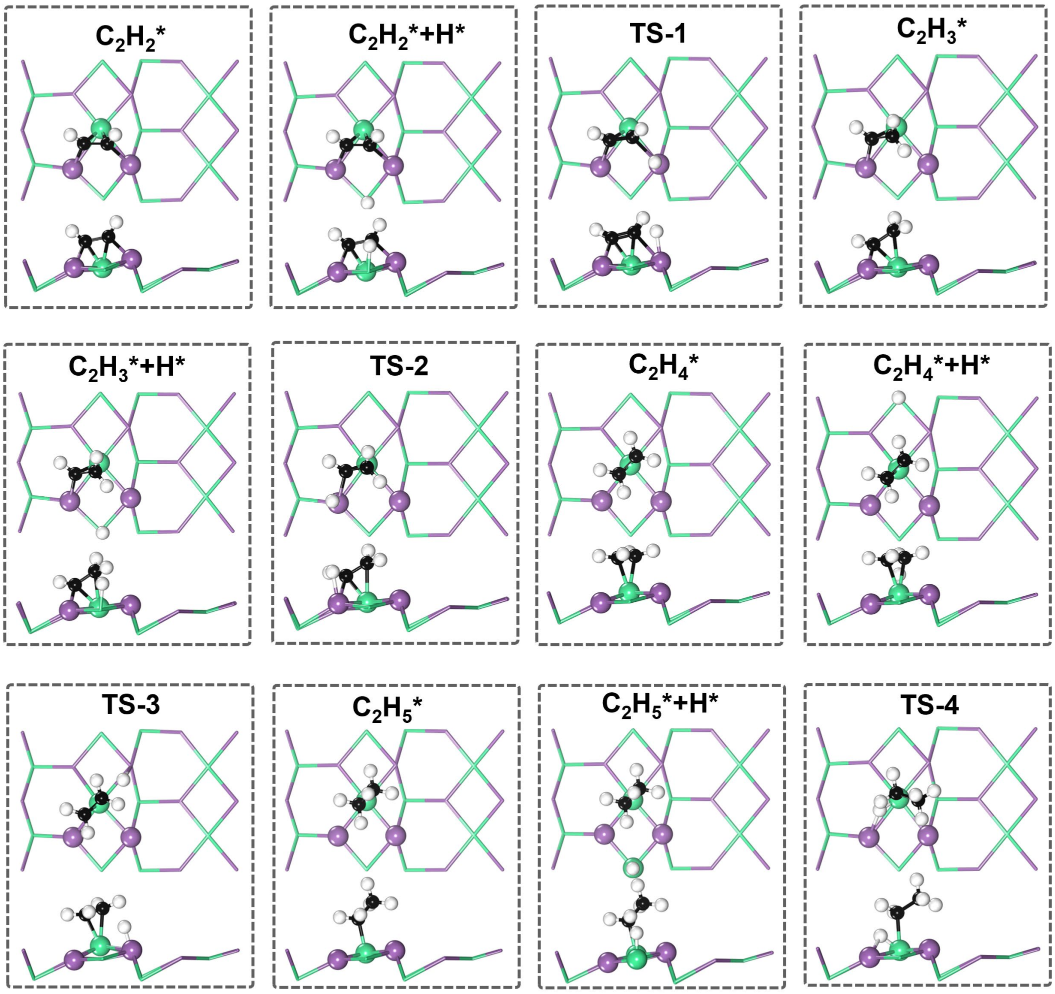

Supplementary Fig. 5. Intermediates on NiSb(101). Configurations of the intermediates involved in the elementary steps of acetylene hydrogenation on the NiSb(101) surface.

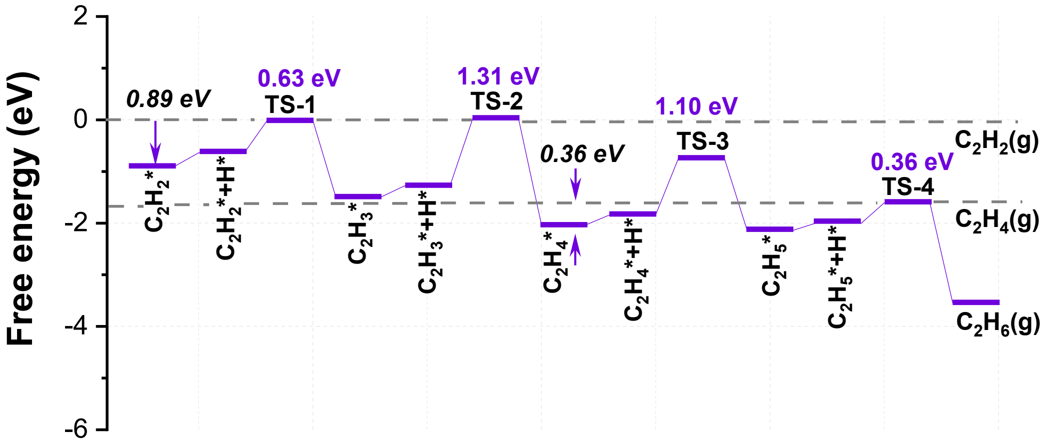

Supplementary Fig. 6. Acetylene hydrogenation on NiSb(102). Free energy profiles for sequential hydrogenation processes of acetylene to ethane on the NiSb(102) surface.

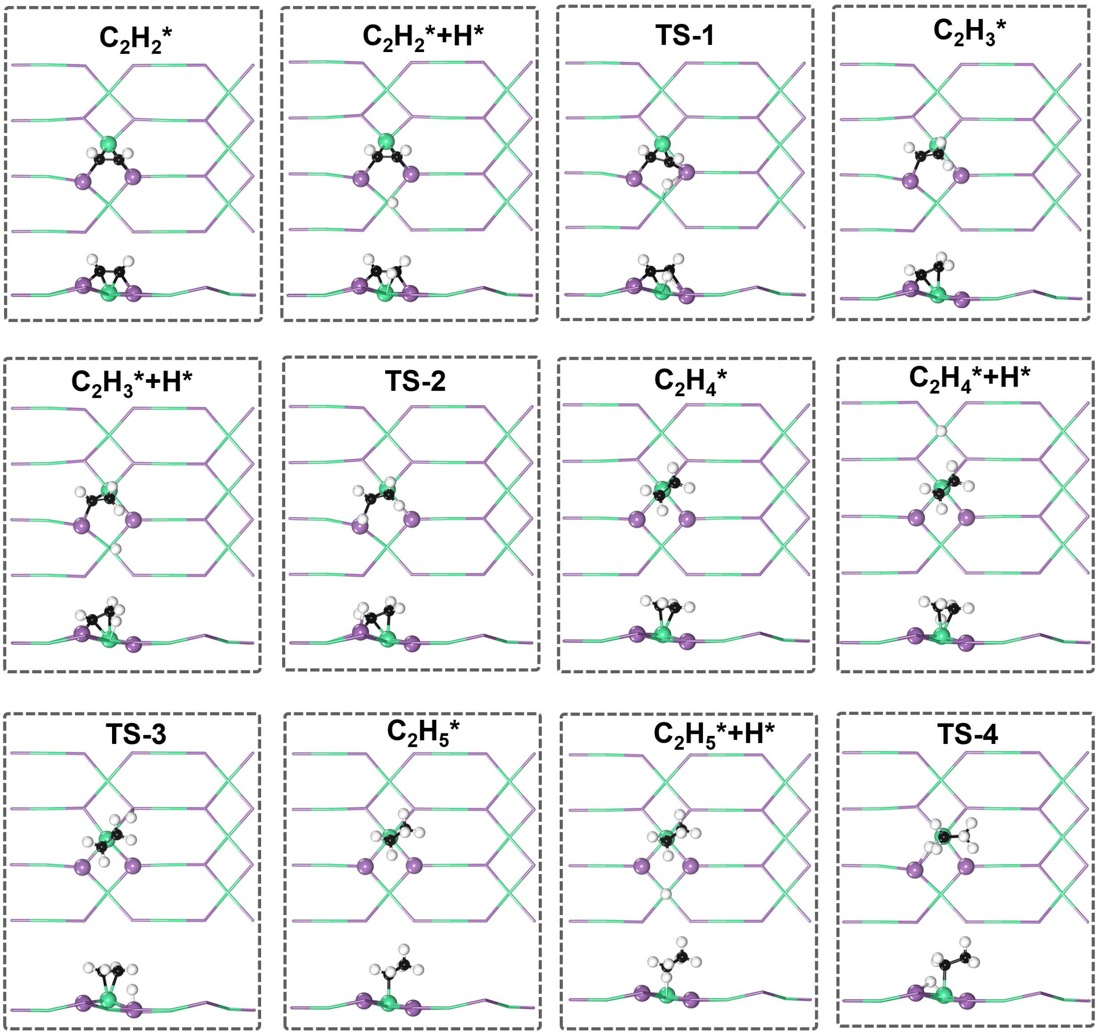

Supplementary Fig. 7. Intermediates on NiSb(102). Configurations of the intermediates involved in the elementary steps of acetylene hydrogenation on the NiSb(102) surface.

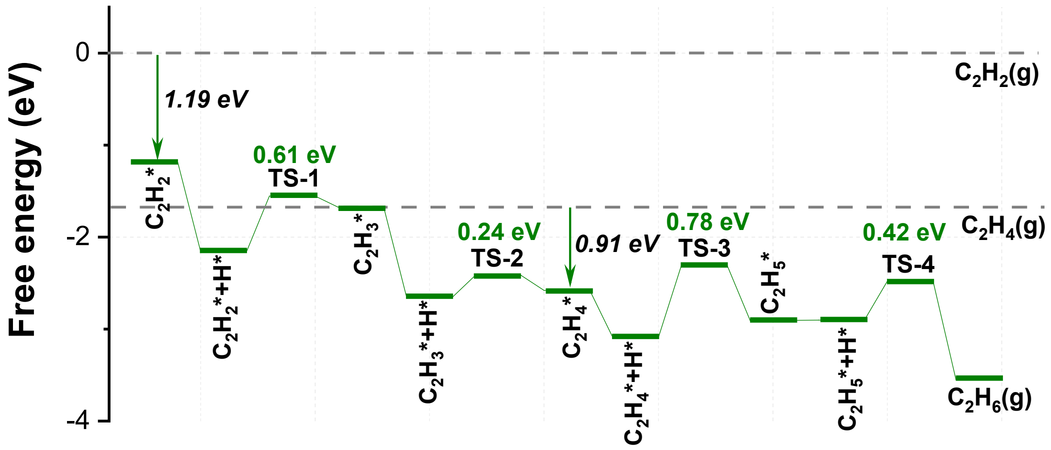

Supplementary Fig. 8. Acetylene hydrogenation on NiSb(100). Free energy profiles for sequential hydrogenation processes of acetylene to ethane on the NiSb(100) surface.

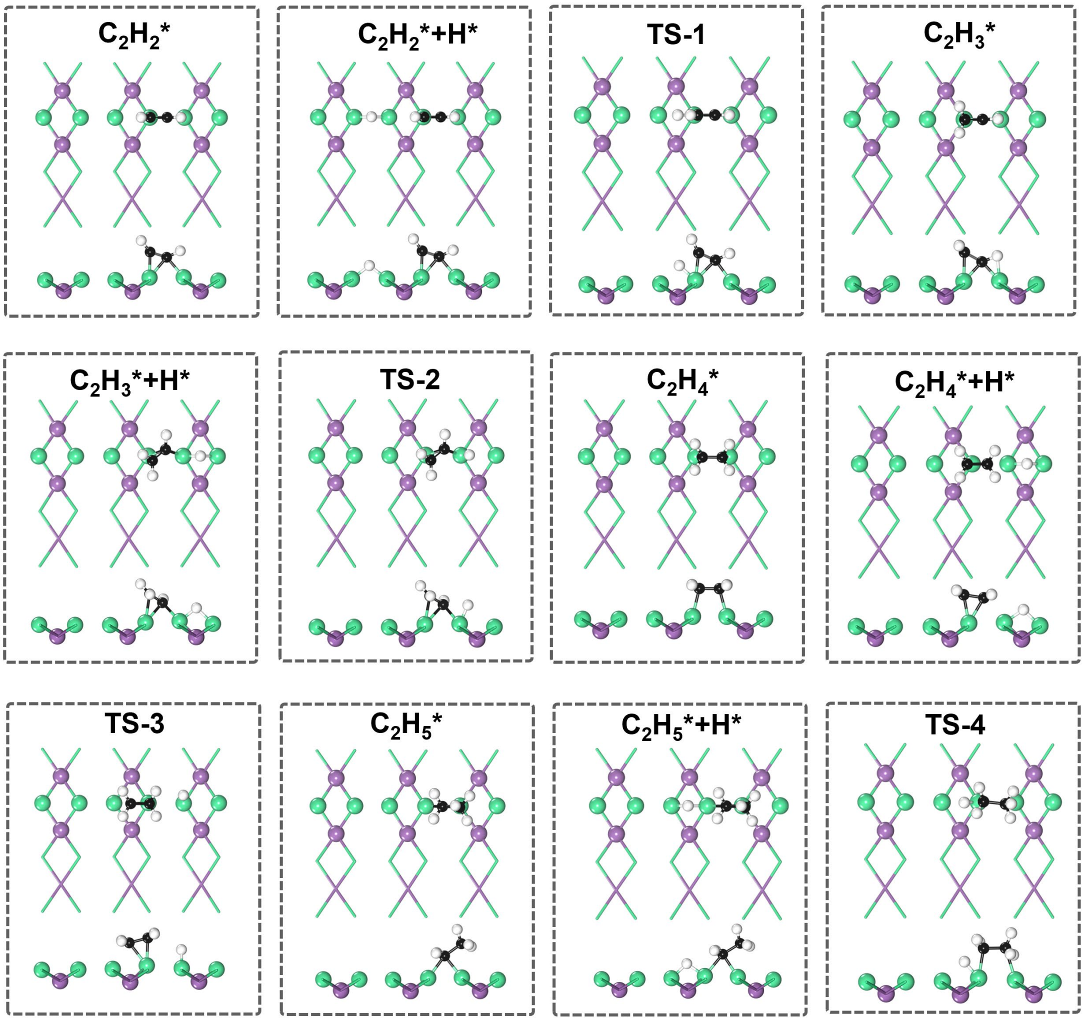

Supplementary Fig. 9. Intermediates on NiSb(100). Configurations of the intermediates involved in the elementary steps of acetylene hydrogenation on the NiSb(100) surface.

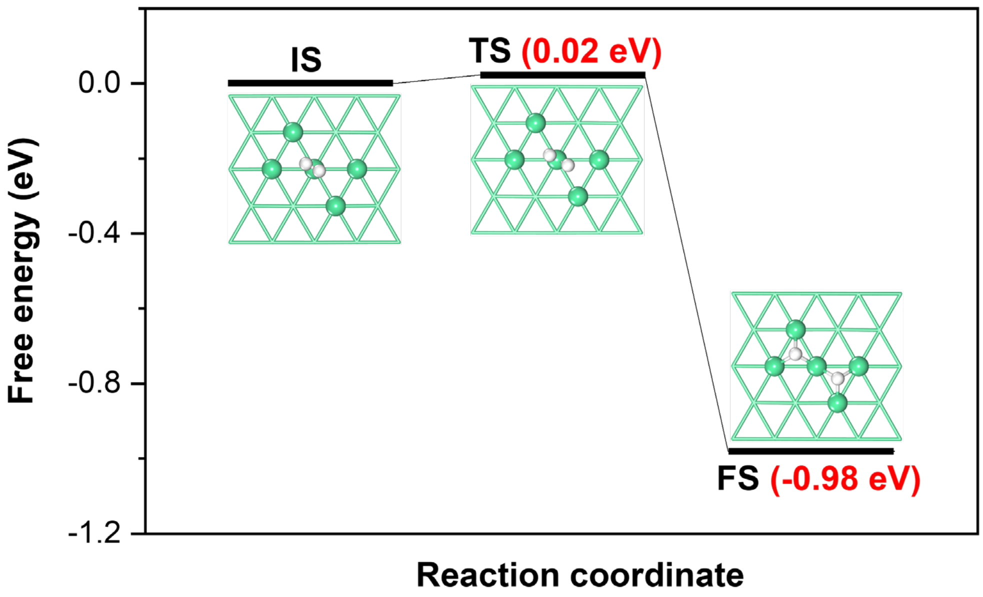

Supplementary Fig. 10. H_2 dissociation on Ni(111). Free energy profile of H_2 dissociation into H atoms on Ni(111) surface.

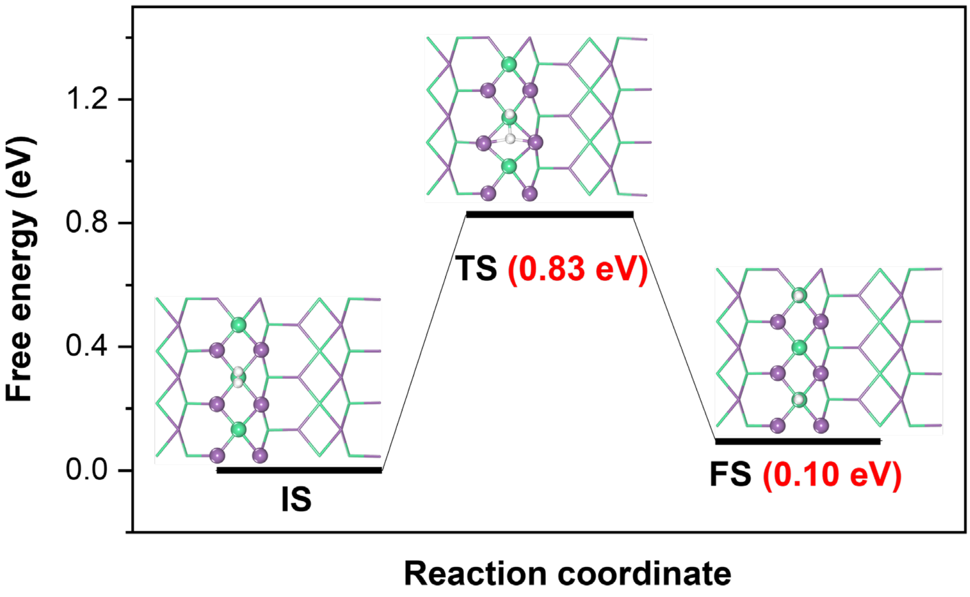

Supplementary Fig. 11. H_2 dissociation on NiSb(101). Free energy profile of H_2 dissociation into H atoms on NiSb(101) surface.

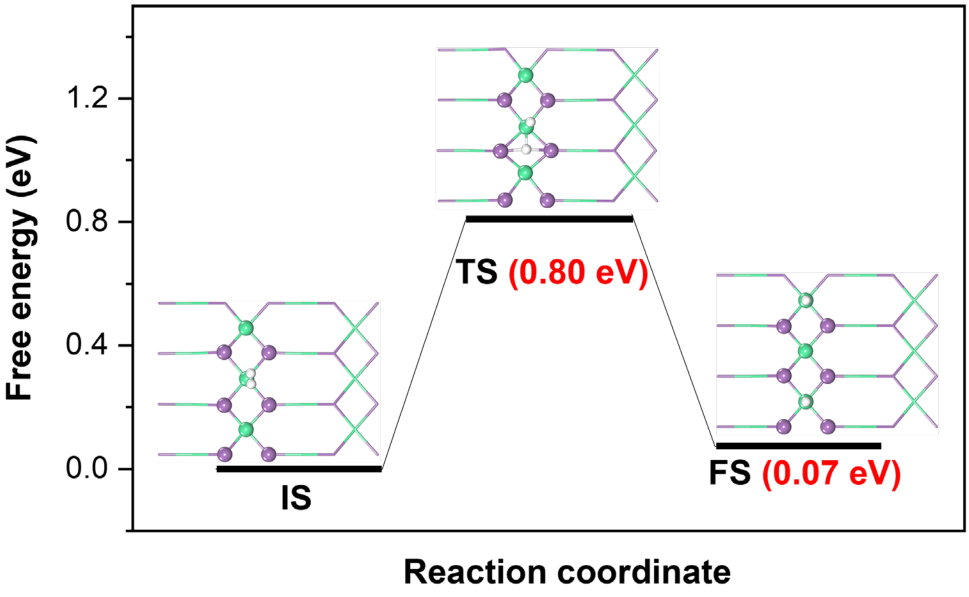

Supplementary Fig. 12. H_2 dissociation on NiSb(102). Free energy profile of H_2 dissociation into H atoms on NiSb(102) surface.

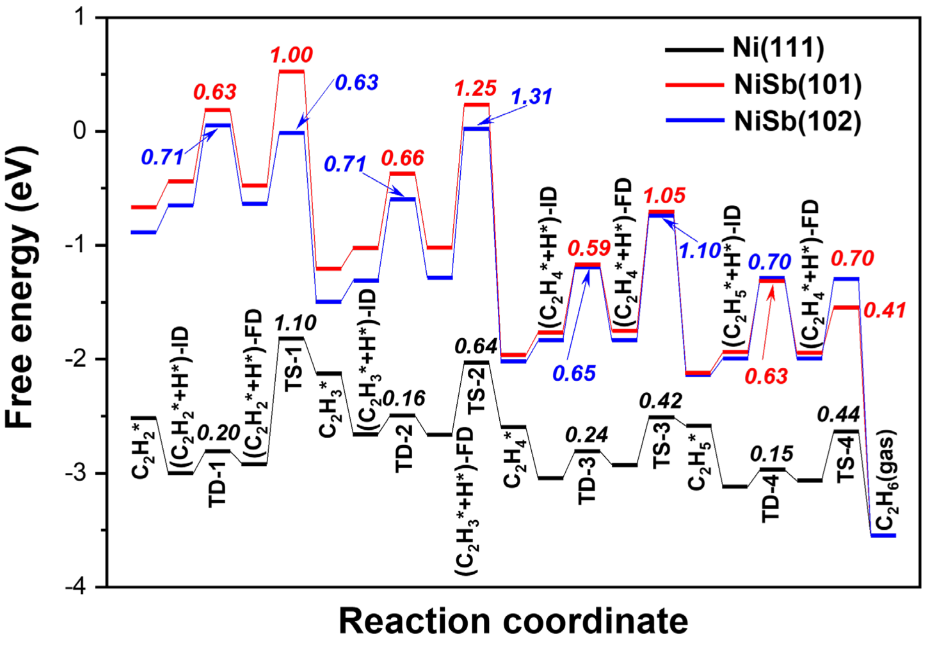

Supplementary Fig. 13. Acetylene hydrogenation involved in H diffusion. Free energy profiles of the overall acetylene hydrogenation reactions over the Ni(111), NiSb(101) and NiSb(102) surfaces.

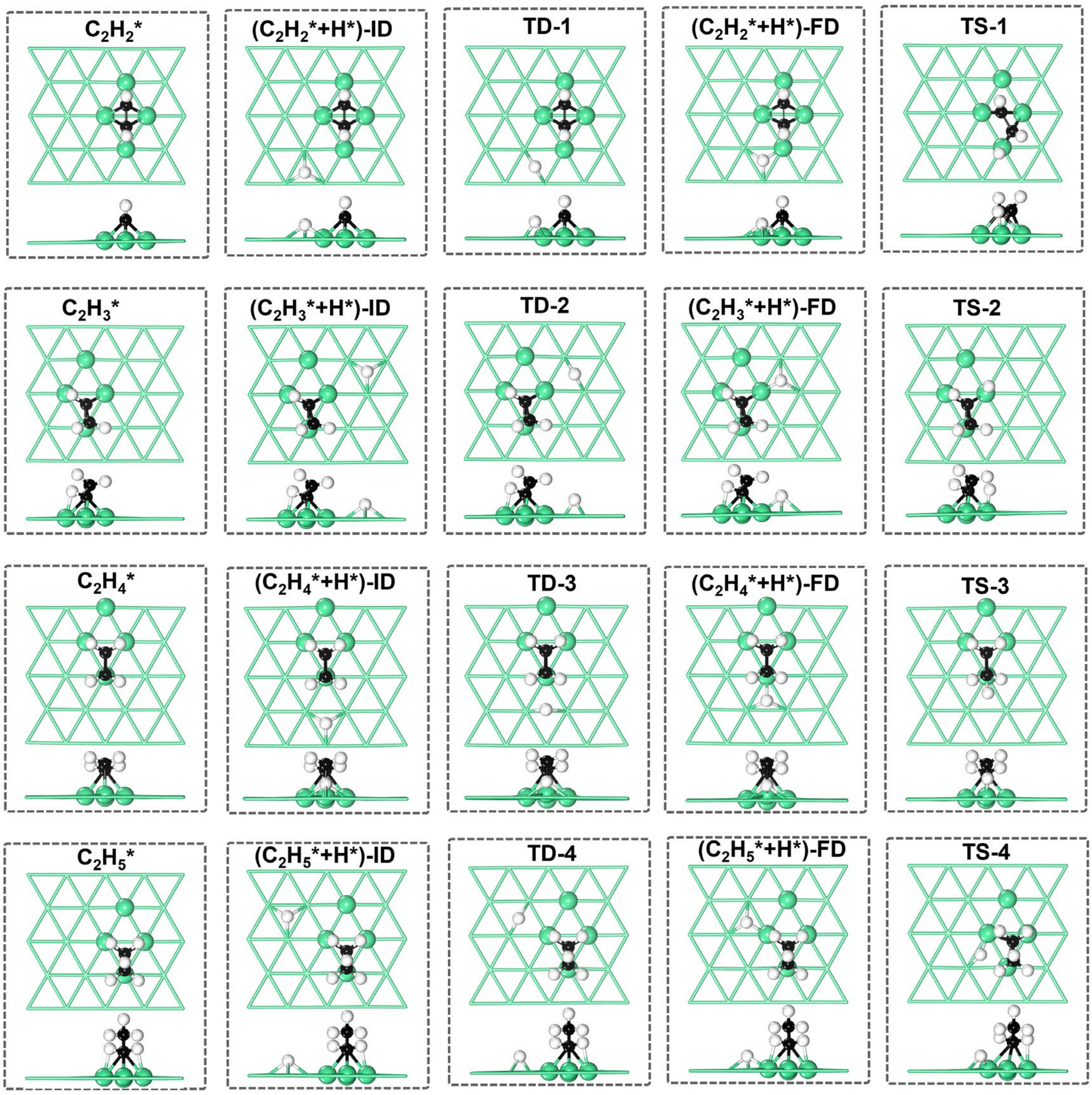

Supplementary Fig. 14. Intermediates on Ni(111). Configurations of intermediates involved in the hydrogen diffusion and acetylene hydrogenation over the Ni(111) surface. “ID”, “TD” and “FD” represent the initial states, transition states and the final states of the diffusion processes, respectively.

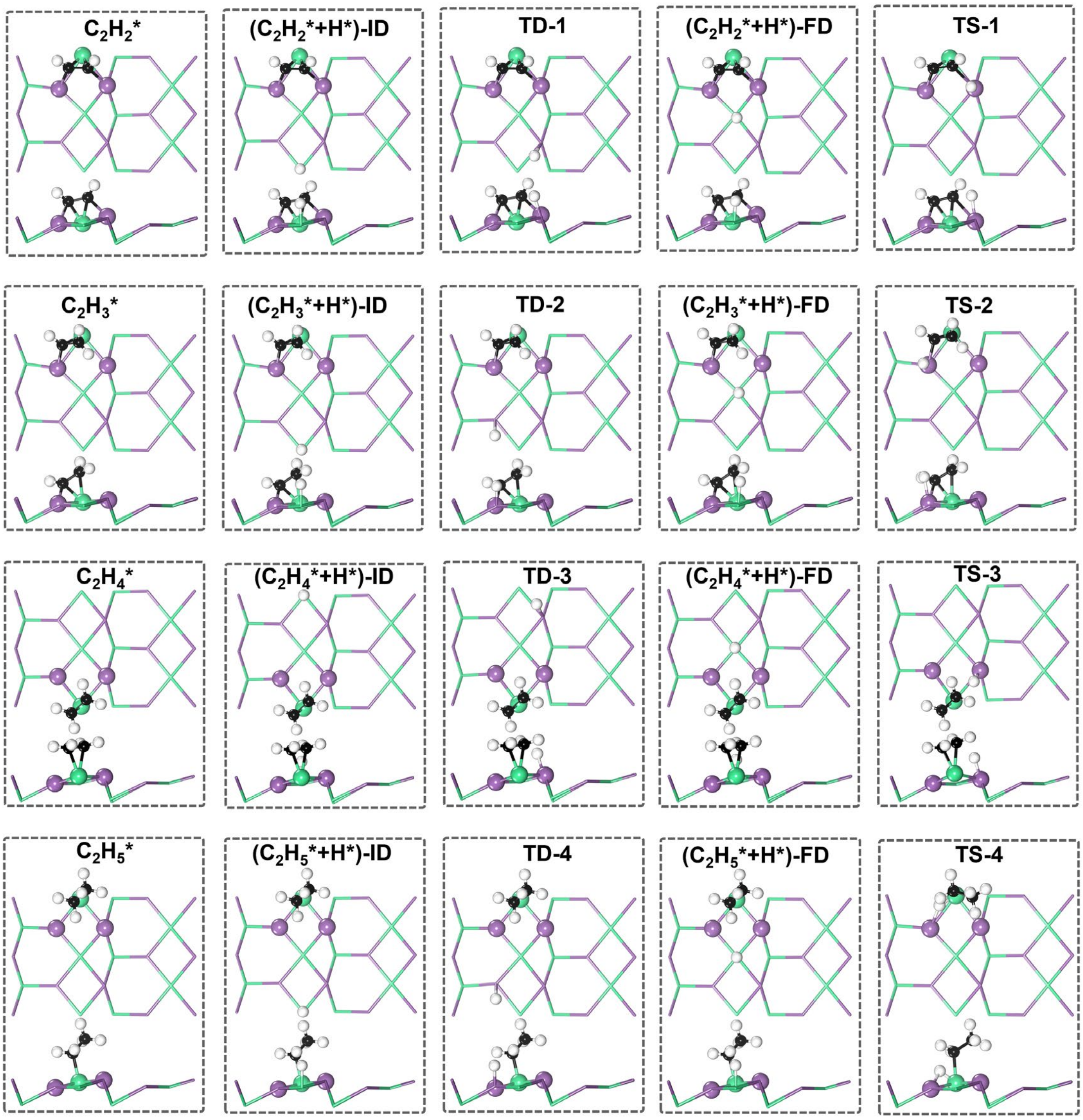

Supplementary Fig. 15. Intermediates on NiSb(101). Configurations of intermediates involved in the hydrogen diffusion and acetylene hydrogenation over the NiSb(101) surface. "ID", "TD" and "FD" represent the initial states, transition states and the final states of the diffusion processes, respectively.

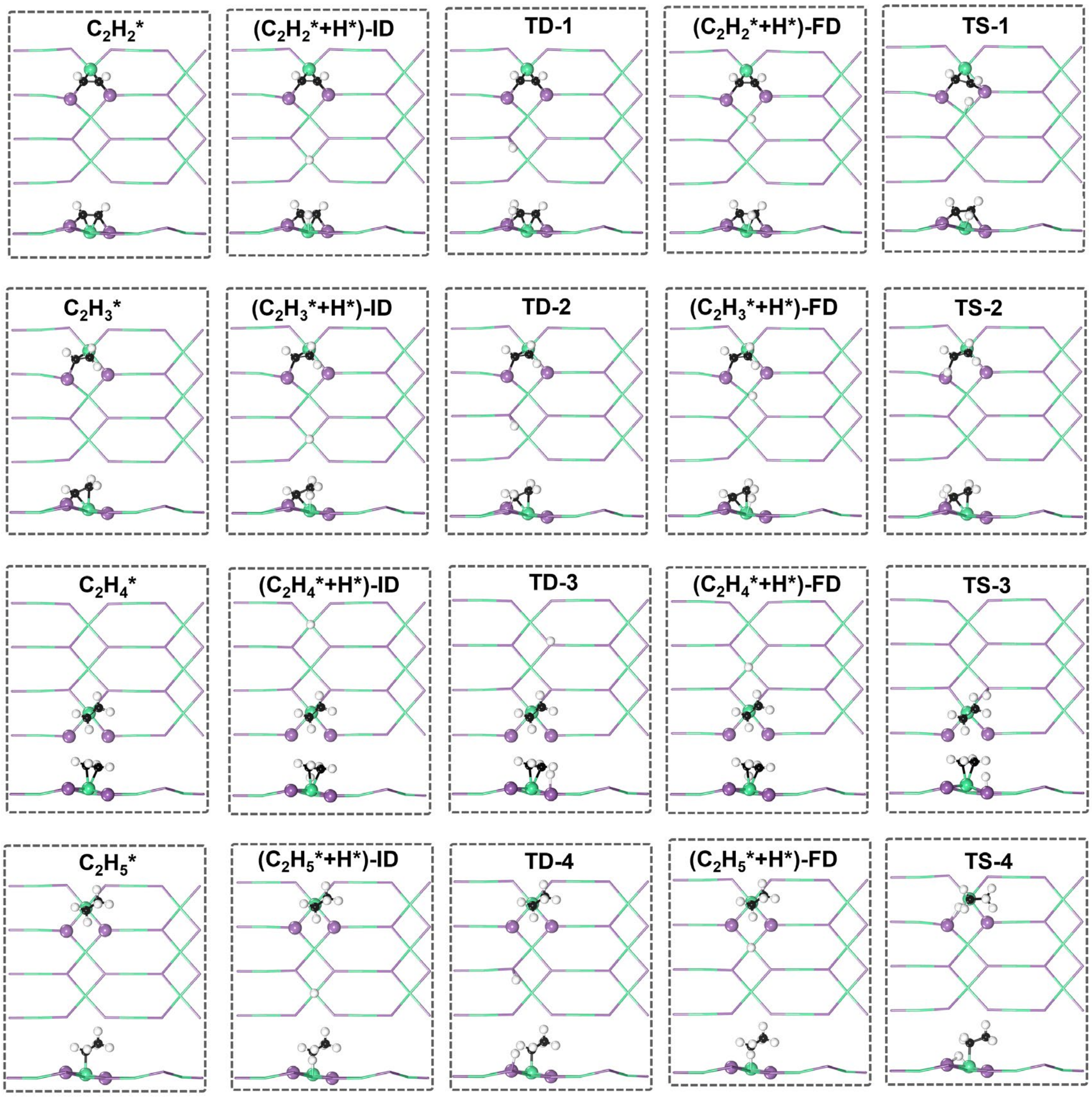

Supplementary Fig. 16. Intermediates on NiSb(102). Configurations of intermediates involved in the hydrogen diffusion and acetylene hydrogenation over the NiSb(102) surface. "ID", "TD" and "FD" represent the initial states, transition states and the final states of the diffusion processes, respectively.

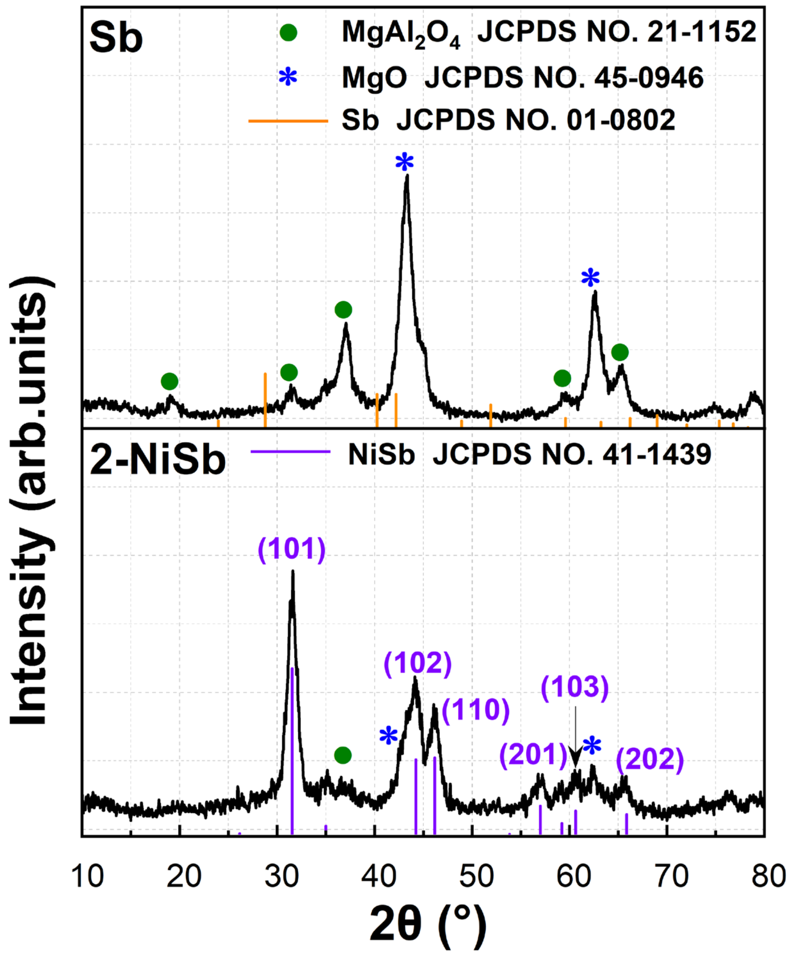

Supplementary Fig. 17. XRD characterizations. XRD spectra of the 2-NiSb and Sb catalysts.

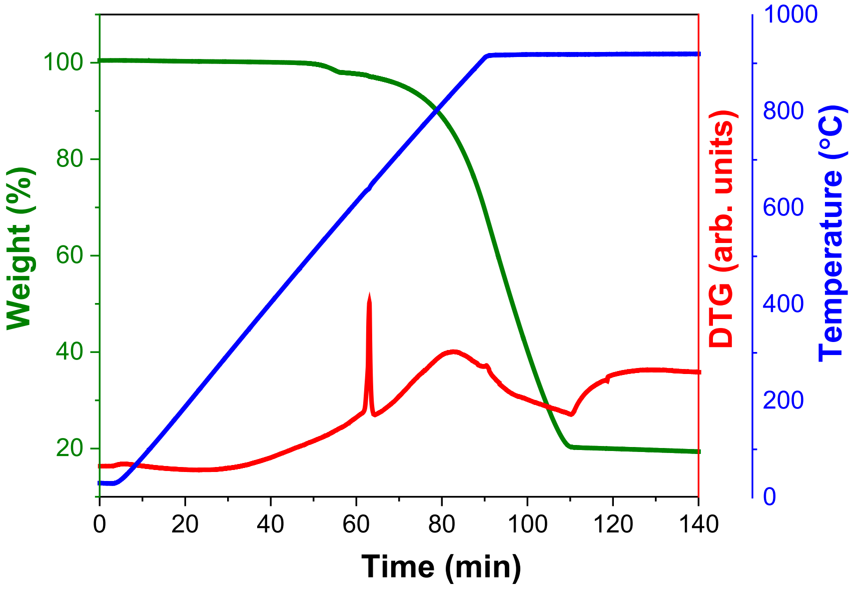

Supplementary Fig. 18. TG-DTG curves of the Sb powder. About 12 mg of the Sb powder was pretreated with 20 vol% H_2/N_2 with a flow rate of 50 mL/min at 120 °C for 30 min, and then cooled to room temperature. Subsequently, the TG-DTG measurement was started with increasing the temperature from room temperature to 900 °C at a rate of 10 °C/min and then maintained at 900 °C for 1 h.

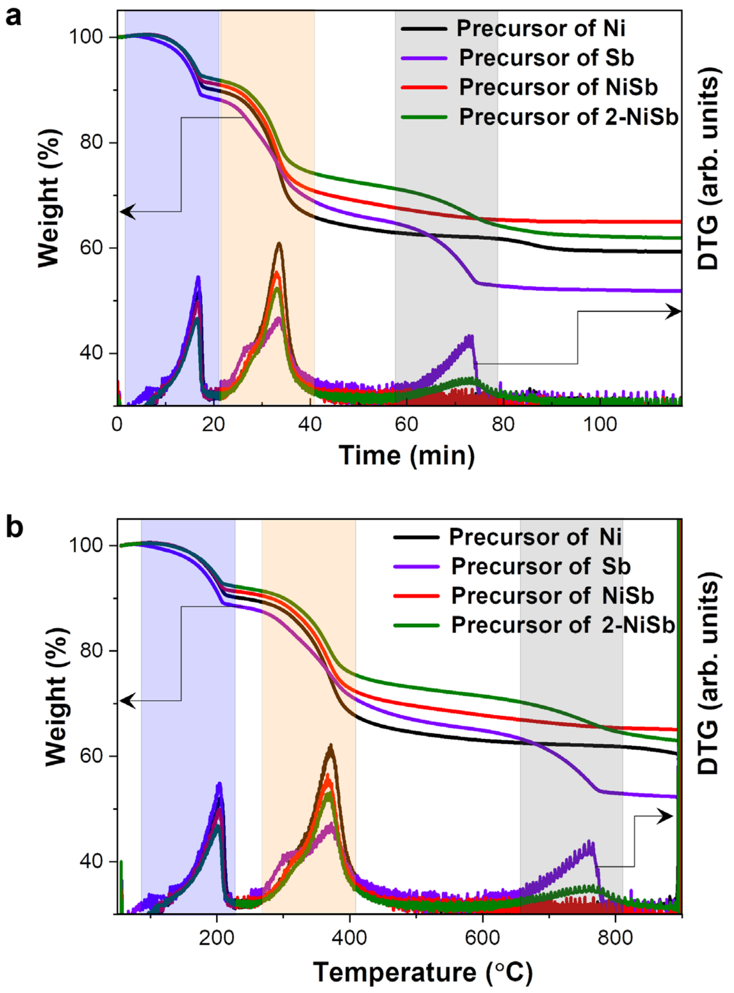

Supplementary Fig. 19. **TG-DTG curves of the precursors of Ni, Sb and NiSb and 2-NiSb.** (a) Time-dependent and (b) temperature-dependent TG-DTG profiles. About 6 mg of the sample was pretreated with 20 vol% H_2/Ar with a flow rate of 50 mL/min at 120 °C for 30 min, and then cooled to room temperature. Subsequently, the TG-DTG measurement was started with increasing the temperature from room temperature to 900 °C at a rate of 10 °C/min and then maintained at 900 °C for 1 h.

To explore the evaporation of Sb in the sample with a nominal ratio of 1:2 (i.e., 2-NiSb) during the thermal process, thermogravimetric analysis measurements under a similar condition used for the thermal process were further performed. The TG-DTG curves of the referred pure Sb powder show that Sb powder begins to melt and vaporize at 630 °C, and the vaporization gradually accelerates after reaching the melting point (Supplementary **Fig. 18**). Notably, the molten Sb is found to easily vaporize at 900 °C, although Sb boils at ~ 1590 °C. The TG-DTG curves of the as-prepared precursors of Ni, Sb, NiSb and 2-NiSb show three weight loss regions (Supplementary **Fig.**

19). The first weight loss region from room temperature to 200 ^{o}C is assigned to the loss of water physically adsorbed on the surface of the LDHs and that trapped in the interlayer of LDHs[3], and the second one from 250 to 410 ^{o}C is attributed to the simultaneous dehydroxylation and decarbonation of the LDHs[3, 4]. The second weight loss, that occurring from 250 to 410 ^{o}C, amounts to about 26%, 21%, 22% and 19% in all the precursor of Ni, Sb, NiSb and 2-NiSb, respectively, close to the value theoretically calculated from its chemical compositions. The third weight loss from 650 to 900 ^{o}C ascribed to the vaporization of extra Sb is observed for the precursors of Sb and 2-NiSb but not for those of Ni and NiSb, indicating facile loss of Sb during the thermal process at the temperature lower than the boil point of Sb.

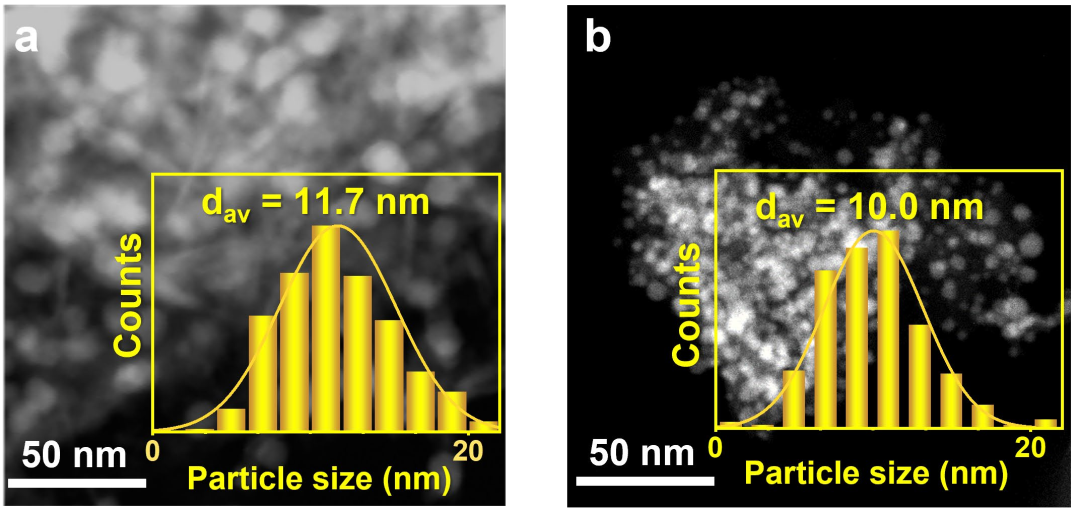

Supplementary Fig. 20. HAADF-STEM images of the (a) Ni and (b) NiSb catalysts. The insets in high-resolution STEM images are the corresponding histograms of the particle size distributions.

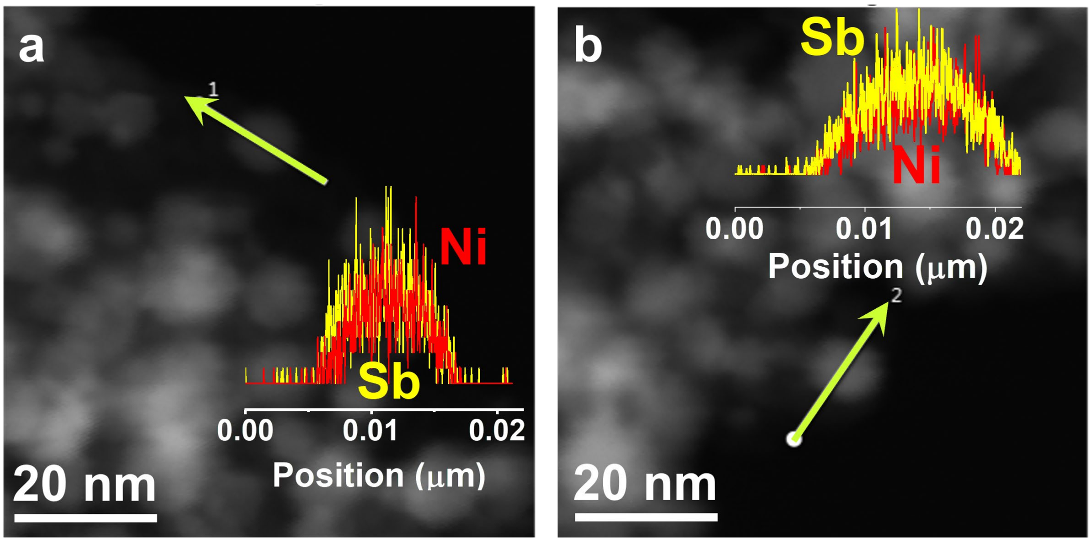

Supplementary Fig. 21. HAADF-STEM images of NiSb catalyst. The insets in regions of (a) and (b) are the corresponding EDS line-scanning profile analysis.

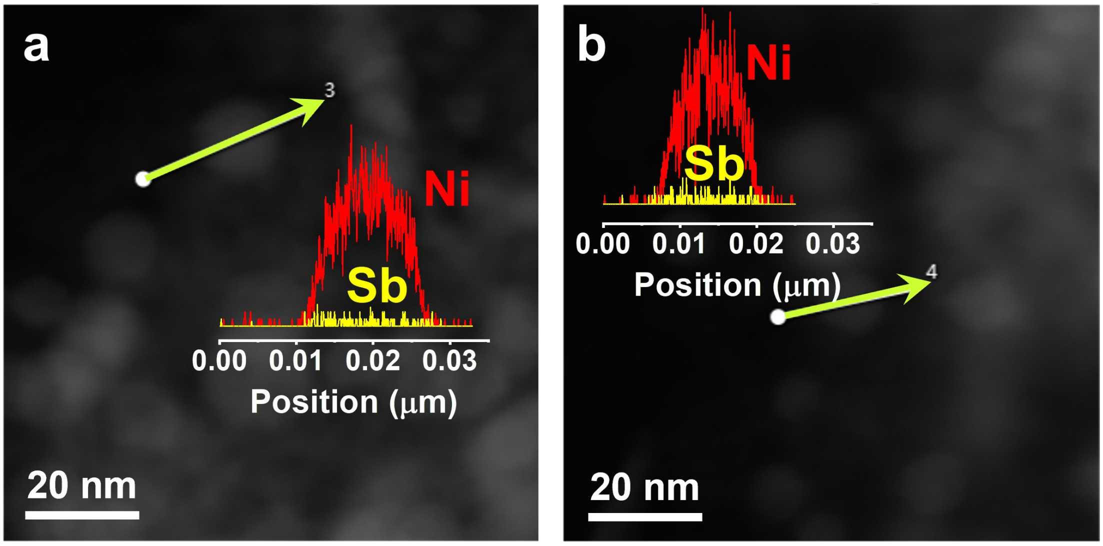

Supplementary Fig. 22. HAADF-STEM images of Ni catalyst. The insets in regions of (a) and (b) are the corresponding EDS line-scanning profile analysis.

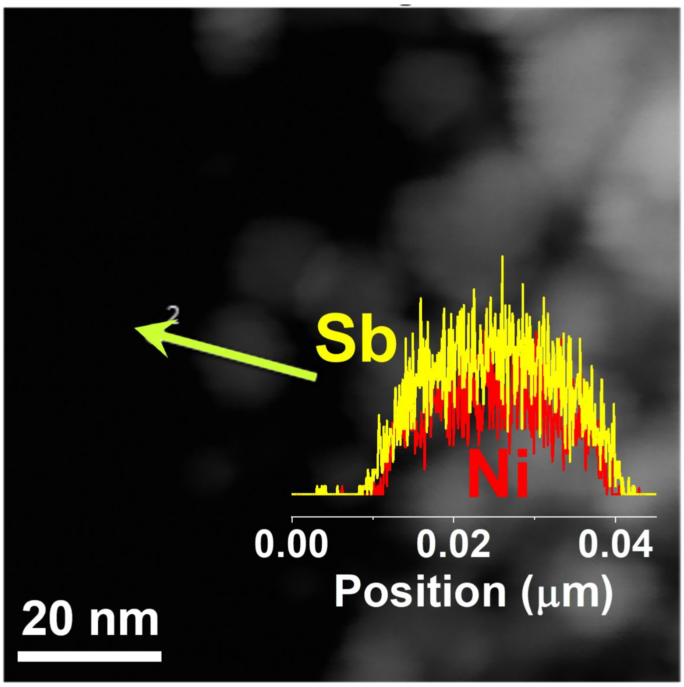

Supplementary Fig. 23. HAADF-STEM image of 2-NiSb catalyst. The inset presented in this high-resolution STEM image is the corresponding EDS line-scanning profile analysis.

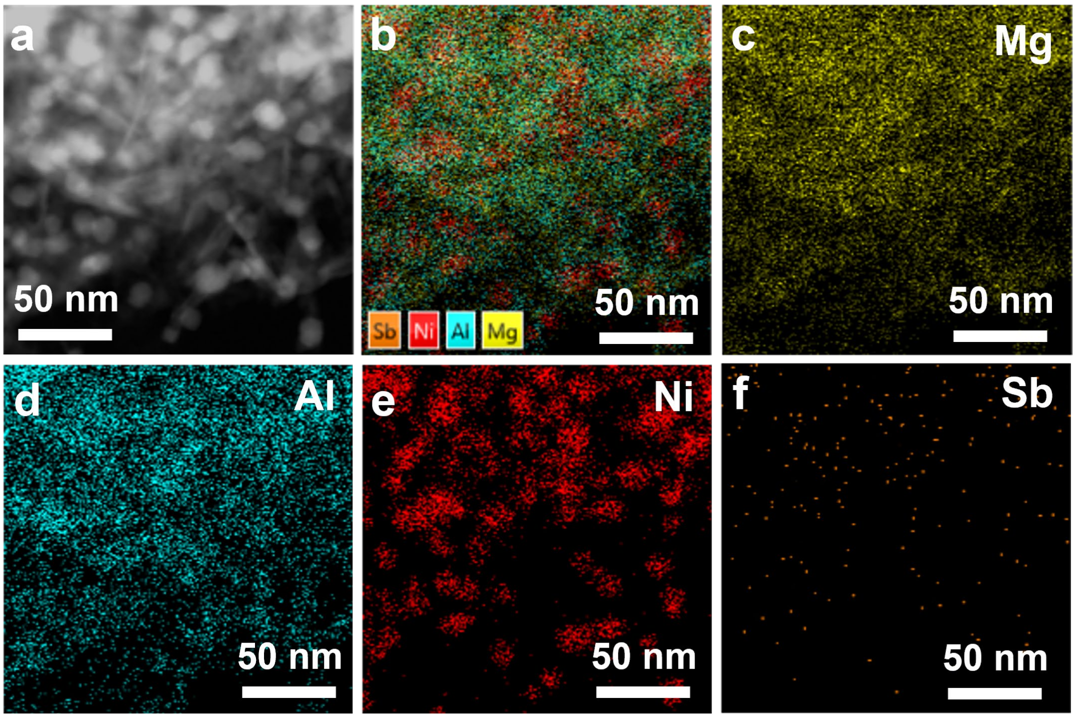

Supplementary Fig. 24. HAADF-STEM images of Ni catalyst. (a) HAADF-STEM image and (b-f) the corresponding EDX mapping of the Ni catalyst.

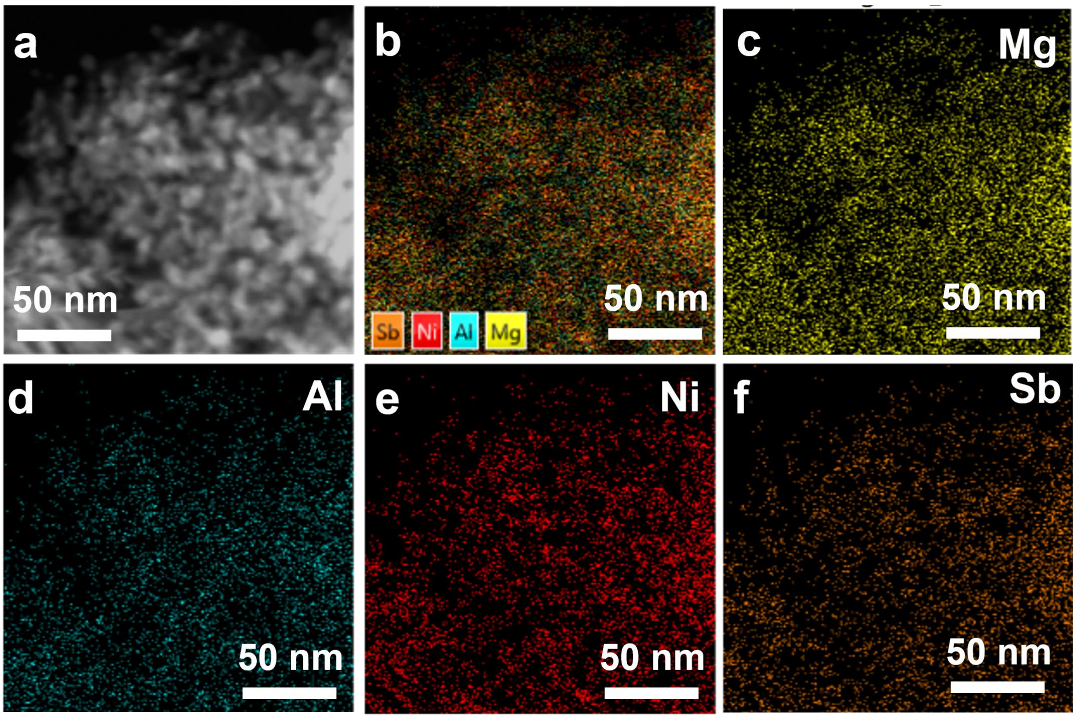

Supplementary Fig. 25. HAADF-STEM images of NiSb catalyst. (a) HAADF-STEM image and (b-f) corresponding EDX mapping analysis of the NiSb catalyst.

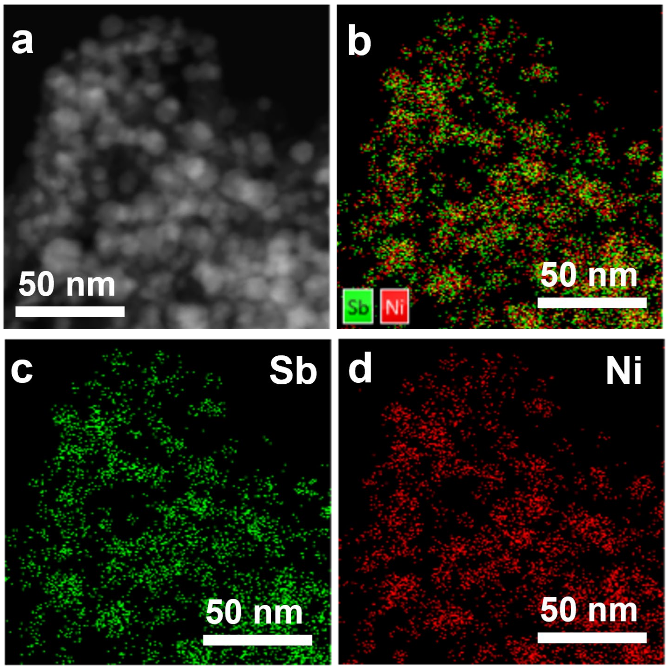

Supplementary Fig. 26. HAADF-STEM images of 2-NiSb catalyst. (a) HAADF-STEM image and (b-d) corresponding EDX mapping analysis of the 2-NiSb catalyst.

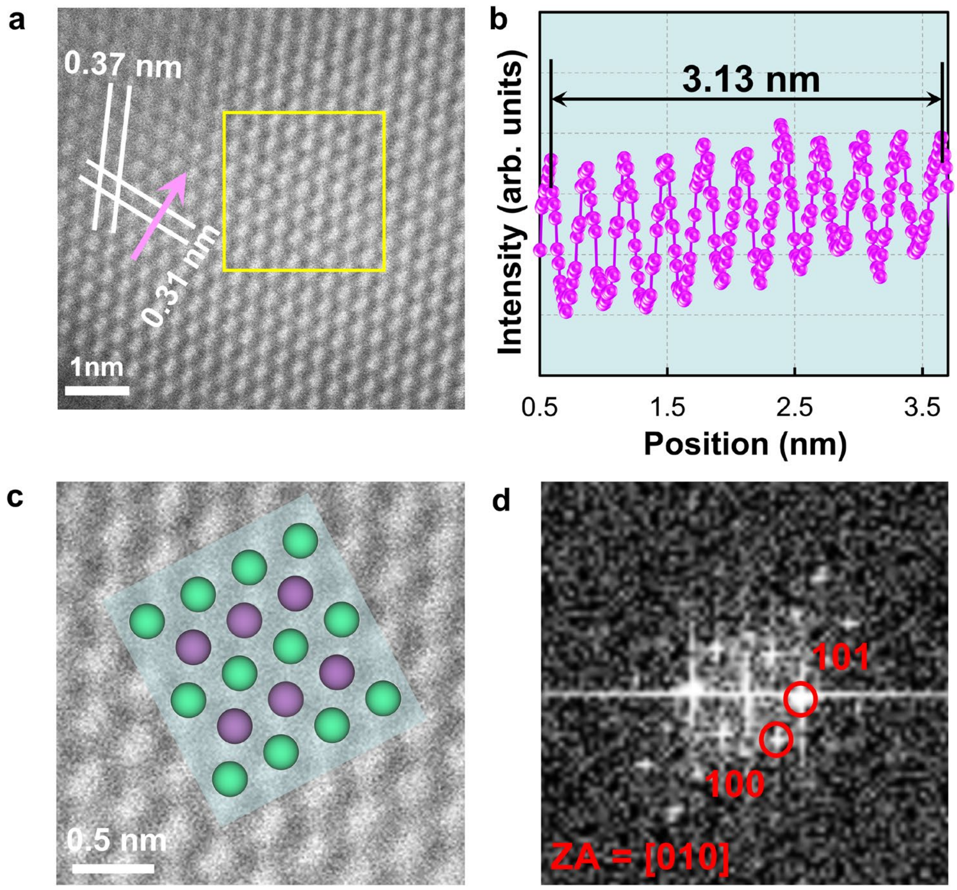

Supplementary Fig. 27. AC-HAADF-STEM images of NiSb catalyst. (a) Representative AC-HAADF-STEM images of the NiSb catalyst. (b) Line intensity profiles along the direct indicated by the violet arrow in (a). (c) Enlarged views of the areas marked by the yellow rectangles in (a) as well as the corresponding crystal models along with [010] zones. (d) The FFT patterns of the NiSb catalyst.

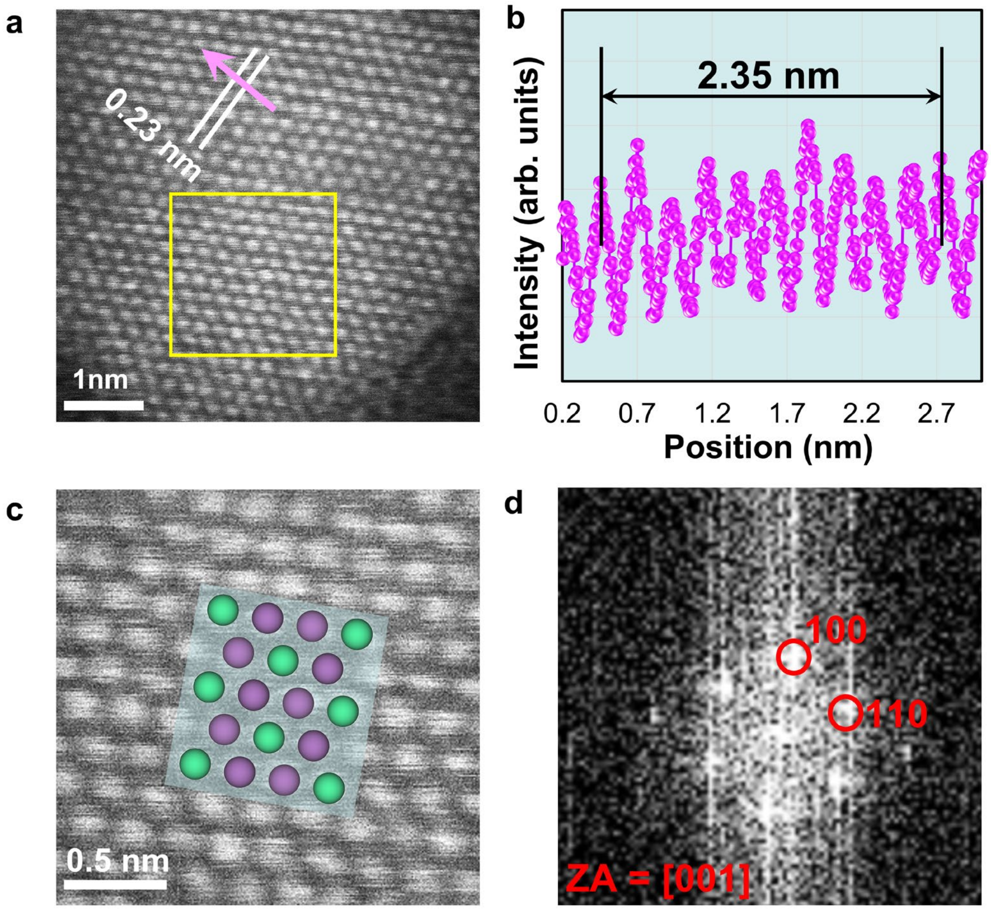

Supplementary Fig. 28. AC-HAADF-STEM images of NiSb catalyst. (a) Representative AC-HAADF-STEM images of the NiSb catalyst. (b) Line intensity profiles along the direct indicated by the violet arrow in (a). (c) Enlarged views of the areas marked by the yellow rectangles in (a) as well as the corresponding crystal models along with [001] zones. (d) The FFT patterns of the NiSb catalyst.

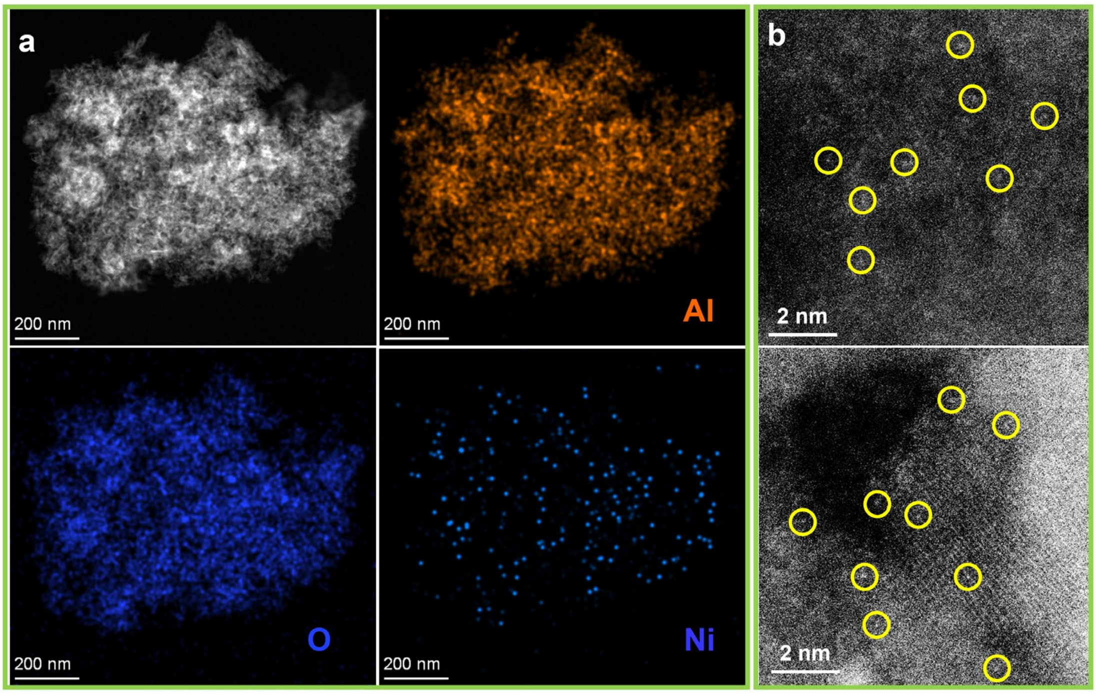

Supplementary Fig. 29. HAADF-STEM images of Ni-SA catalyst. (a) Typical HAADF-STEM image of the Ni-SA catalyst and corresponding EDS mapping images. (b) AC-HAADF-STEM images of Ni-SA at high magnification.

Single-atom Ni catalyst supported on Al_2O_3 (denoted as Ni-SA catalyst) was further synthesized by atomic layer deposition (ALD) technology to compare with the NiSb catalyst featuring with trimer Ni_1Sb_2 sites. The atomic-resolution electron microscopy measurements for the Ni-SA catalyst demonstrate that atomically dispersed Ni on Al_2O_3 without legible presence of cluster or nanoparticles (Supplementary **Fig. 29**). The catalytic performance tests were performed for the Ni-SA catalyst under the same conditions as those for the NiSb catalyst. The Ni-SA catalyst exhibit only 9.2% of acetylene conversion with 72.6% of ethylene selectivity, 16.1% of ethane selectivity and 11.3% of C_4 selectivity at 200 ^{o}C (Supplementary **Fig. 36**), which are inferior to those of the NiSb catalyst. As shown in Supplementary **Fig. 37**, the calculated reaction rate for acetylene conversion on the Ni-SA catalyst is clearly lower than that on the NiSb catalyst, indicating the advantages of the trimer Ni_1Sb_2 sites.

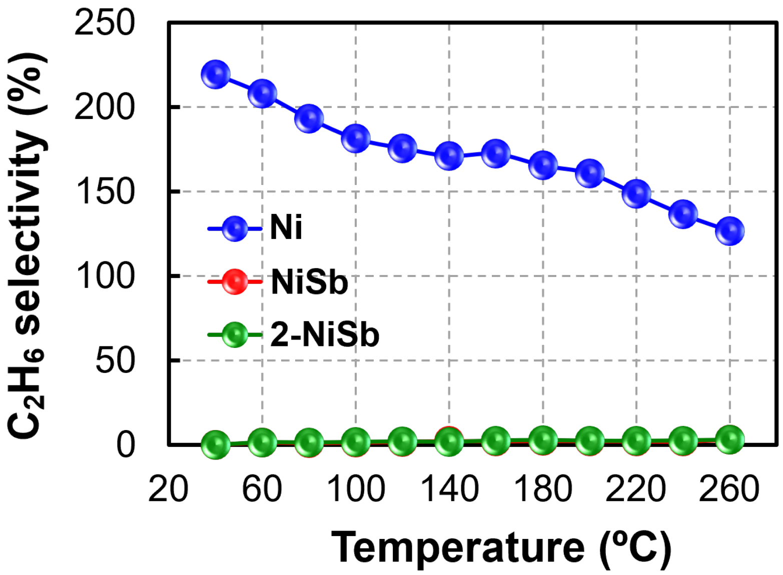

Supplementary Fig. 30. Comparison for C_2H_6 selectivity. C_2H_6 selectivity as a function of reaction temperature over the Ni, NiSb and 2-NiSb catalysts.

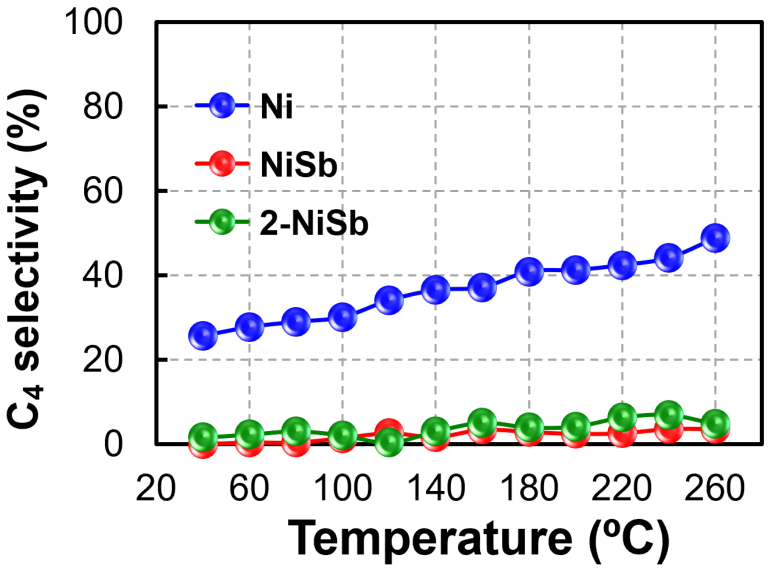

Supplementary Fig. 31. Comparison for C_4 selectivity. C_4 selectivity as a function of reaction temperature over the Ni, NiSb and 2-NiSb catalysts.

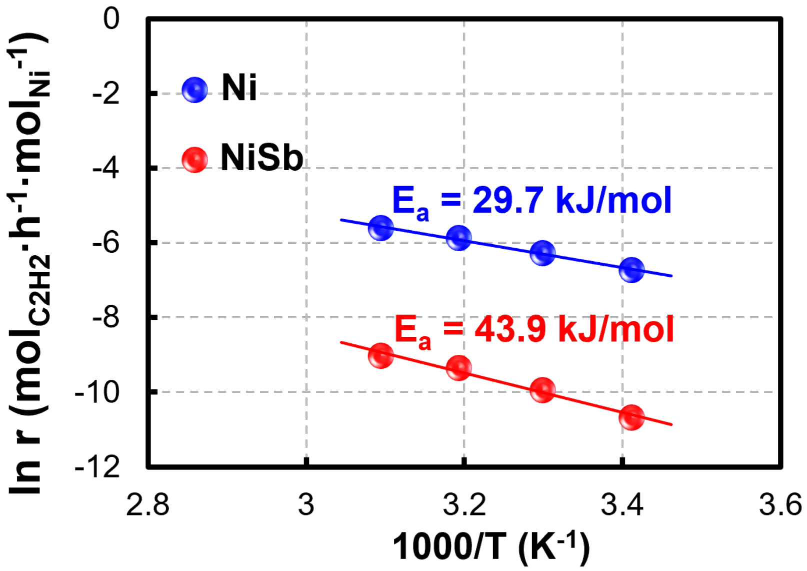

Supplementary Fig. 32. Apparent activation energy for acetylene conversion. Arrhenius plots for acetylene conversion over Ni and NiSb under a 0.5 vol% C_2H_2, 2.5 vol% H_2, 30 vol% C_2H_4, N_2 as balance, the flow rate = 40 mL/min.

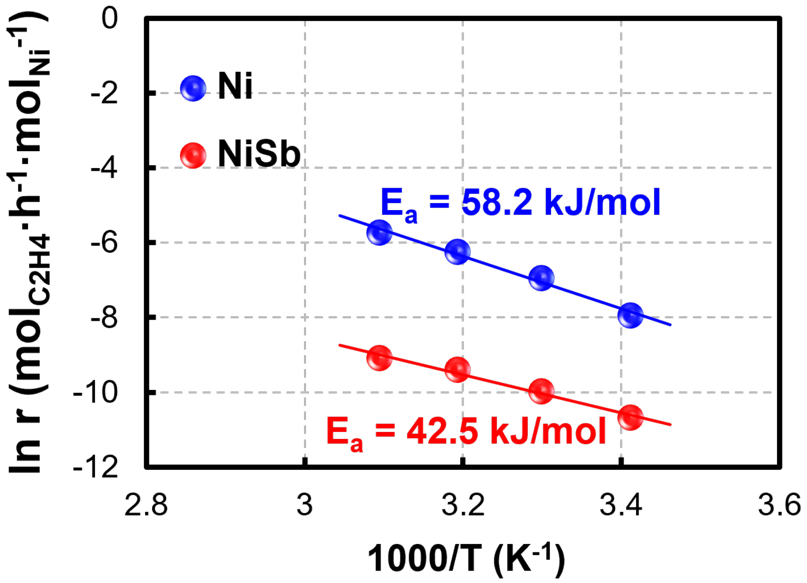

Supplementary Fig. 33. Apparent activation energy for ethylene formation. Arrhenius plots for ethylene formation over Ni and NiSb under a 0.5 vol% C_2H_2, 2.5 vol% H_2, 30 vol% C_2H_4, N_2 as balance, the flow rate = 40 mL/min.

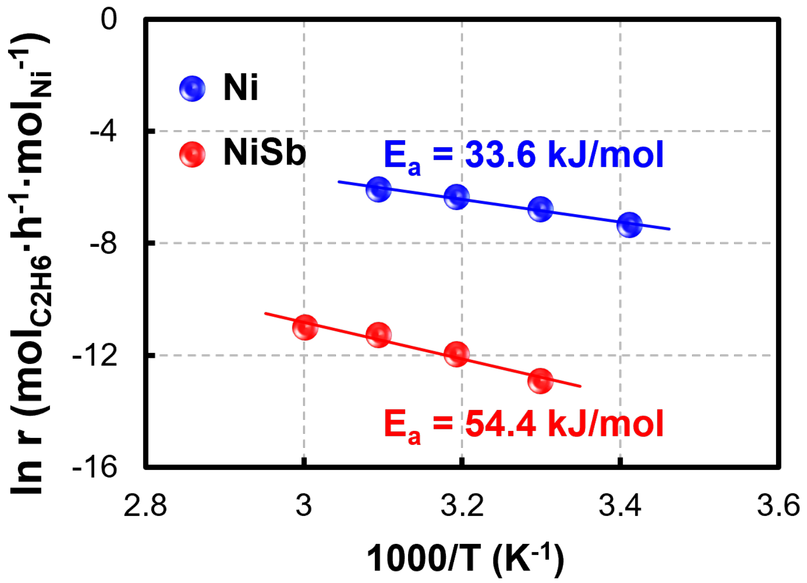

Supplementary Fig. 34. Apparent activation energy for ethane formation. Arrhenius plots for ethane formation over Ni and NiSb under a 2.5 vol% H_2, 30 vol% C_2H_4, N_2 as balance, the flow rate = 50 mL/min.

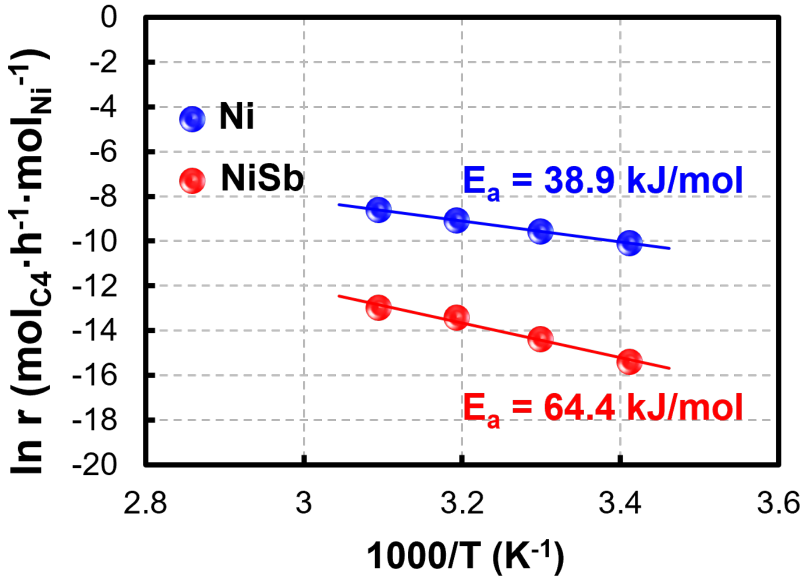

Supplementary Fig. 35. Apparent activation energy for C_4 formation. Arrhenius plots for C_4 formation over Ni and NiSb under a 0.5 vol% C_2H_2, 2.5 vol% H_2, 30 vol% C_2H_4, N_2 as balance, the flow rate = 40 mL/min.

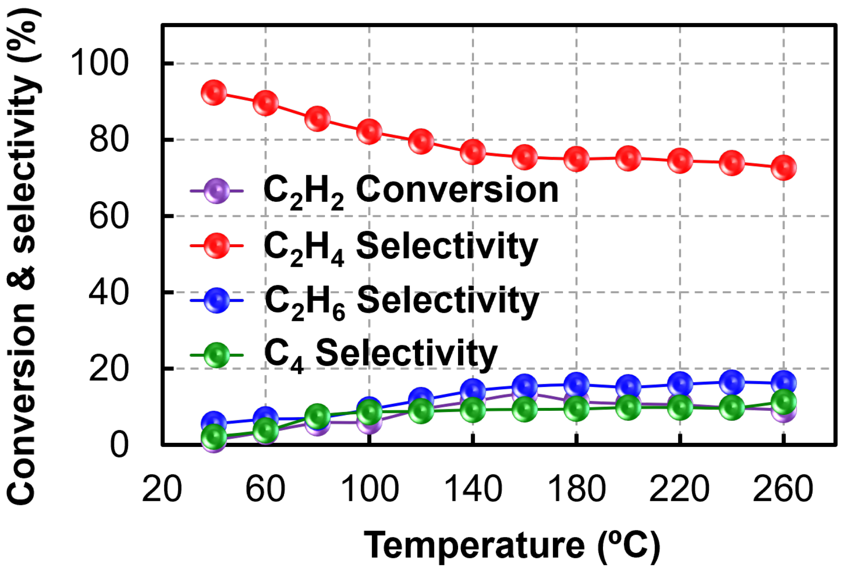

Supplementary Fig. 36. Catalytic performances of Ni-SA catalyst. C_2H_2 conversion, C_2H_4 selectivity, C_2H_6 selectivity and C_4 selectivity as a function of reaction temperature over the Ni-SA catalysts.

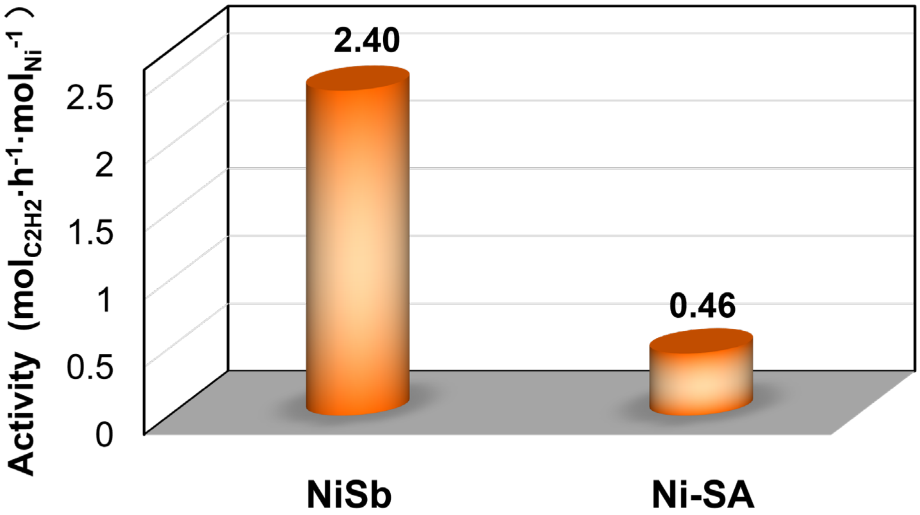

Supplementary Fig. 37. Activity of NiSb and Ni-SA for acetylene hydrogenation. (T = 40 °C; at below 20% acetylene conversion, in the kinetic region).

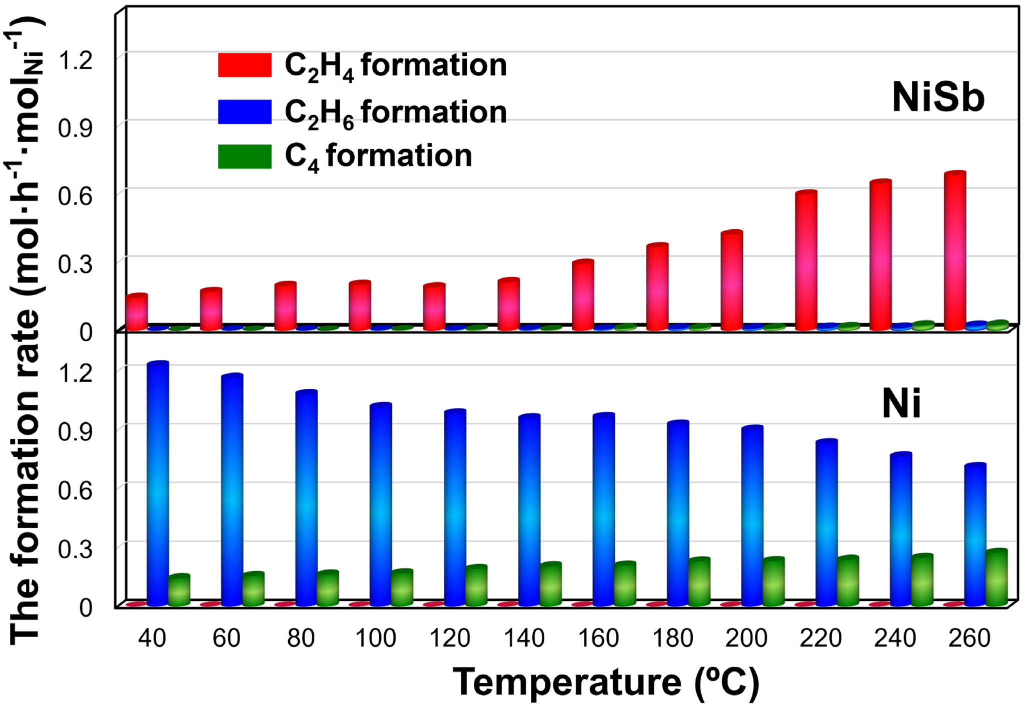

Supplementary Fig. 38. Comparison for formation rates over Ni and NiSb catalysts. C_2H_4, C_2H_6 and C_4 as a function of reaction temperature over the Ni and NiSb catalysts.

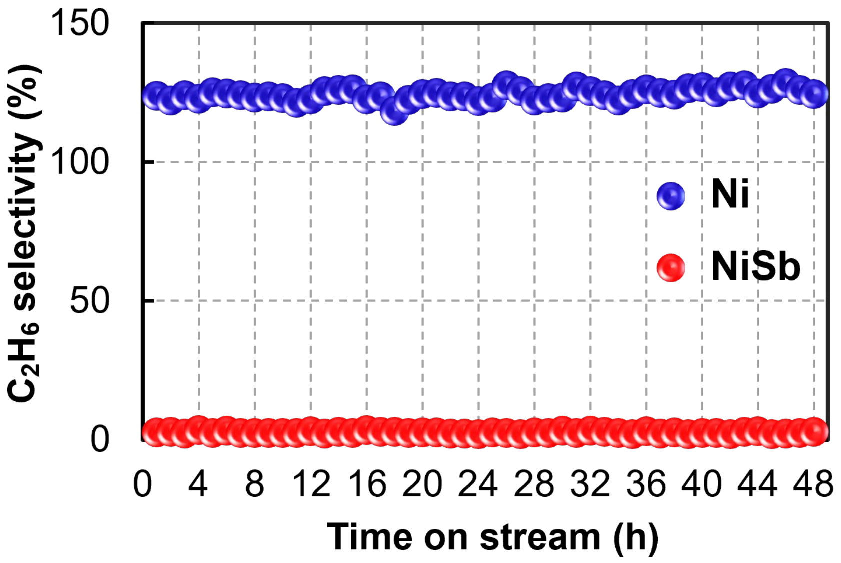

Supplementary Fig. 39. Ethane selectivity with time on stream over Ni and NiSb catalysts. Ethane selectivity determined in the stability test at 240 °C under the conditions: 0.5 vol% C_2H_2, 2.5 vol% H_2, 30 vol% C_2H_4 and balance N_2, flow rate = 30 mL/min.

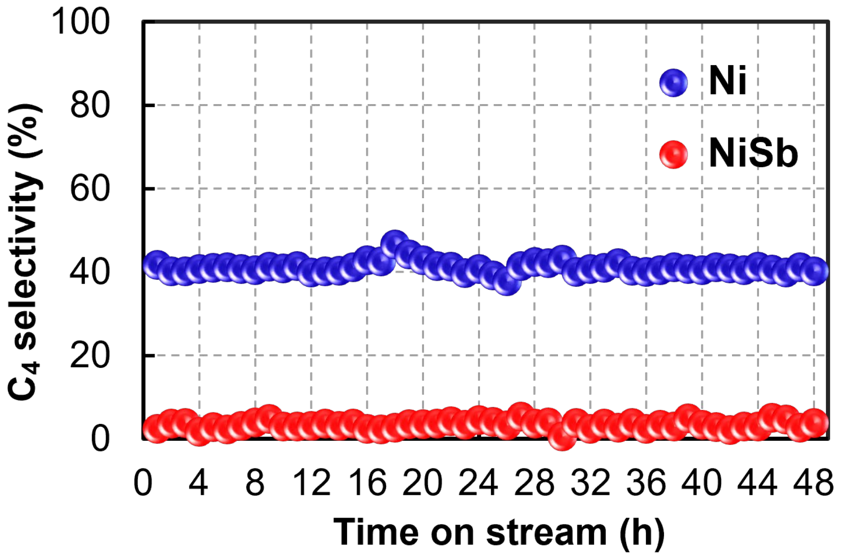

Supplementary Fig. 40. C_4 selectivity with time on stream over Ni and NiSb catalysts. C_4 selectivity determined in the stability test at 240 °C under the conditions: 0.5 vol% C_2H_2, 2.5 vol% H_2, 30 vol% C_2H_4 and balance N_2, flow rate = 30 mL/min.

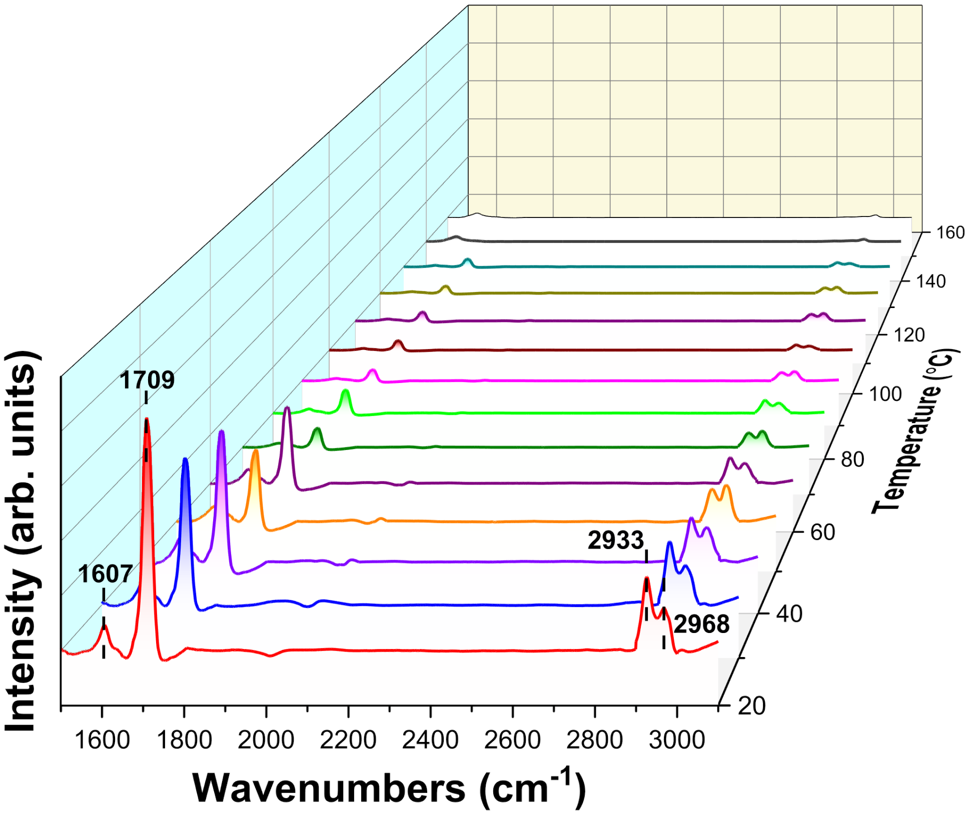

Supplementary Fig. 41. In-situ DRIFTS spectra of Ni catalyst. In-situ DRIFTS spectra for the hydrogenation of the pre-adsorbed acetylene at different temperatures on the Ni catalyst.

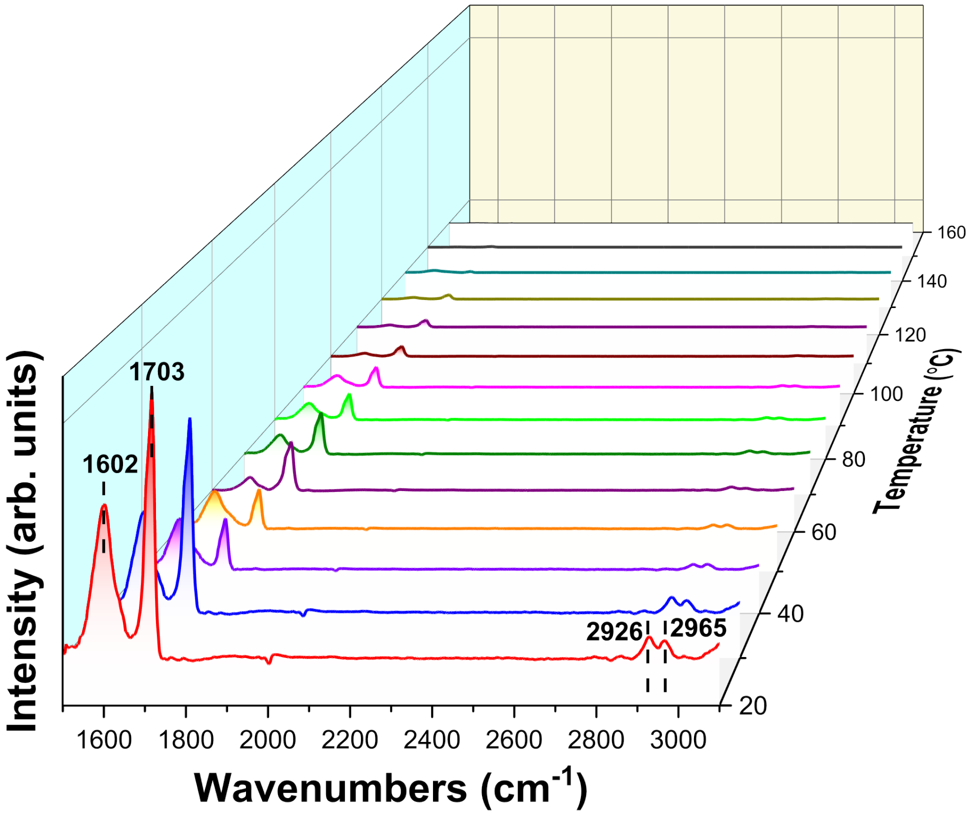

Supplementary Fig. 42. In-situ DRIFTS spectra of NiSb catalyst. In-situ DRIFTS spectra for the hydrogenation of the pre-adsorbed acetylene at different temperatures on the NiSb intermetallic catalyst.

Supplementary Table 1. The percentage of exposed surfaces from Wulff construction of Ni crystallite.

Surfaces	111	110	100
Percentage (%)	73.0	10.1	16.9

Supplementary Table 2. The percentage of exposed surfaces from Wulff construction of NiSb crystallite.

Surfaces	101	102	100
Percentage (%)	55.8	36.2	8.00

Supplementary Table 3. Adsorption energies (E_{ads}/eV) and adsorption free energies (G_{ads}/eV) of C_2H_2 on the Ni(111) surface.

Top view	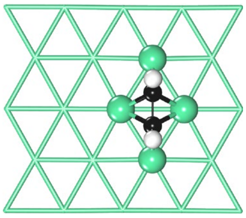	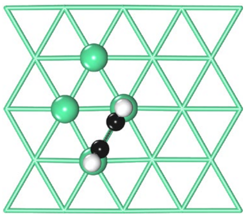	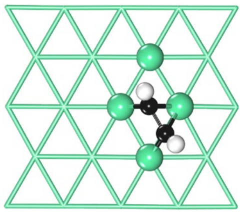
Side view	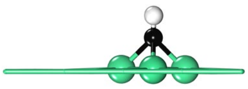	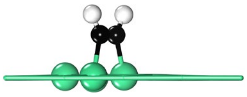	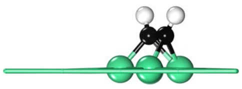
E_{ads} (eV)	-2.75	-1.62	-2.36
G_{ads} (eV)	-2.51	-1.37	-2.11

Supplementary Table 4. Adsorption energies (E_{ads}/eV) and adsorption free energies (G_{ads}/eV) of C_2H_4 on the Ni(111) surface.

Top view	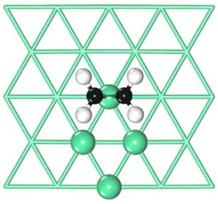	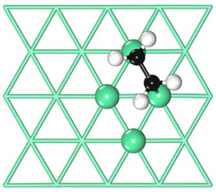	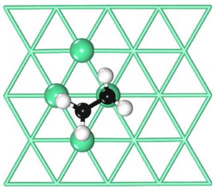	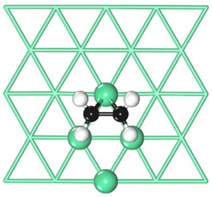
Side view	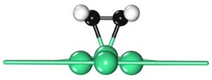	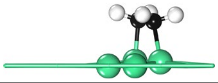	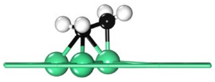	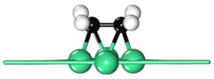
E_{ads} (eV)	-0.92	-0.84	-1.00	-0.93
G_{ads} (eV)	-0.81	-0.74	-0.93	-0.87

Supplementary Table 5. Adsorption energies (E_{ads}/eV) and adsorption free energies (G_{ads}/eV) of C_2H_2 on the NiSb(101) surface.

Top view	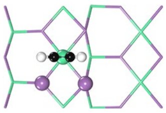	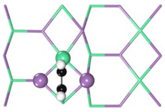	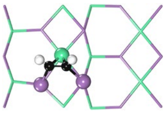	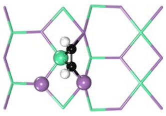	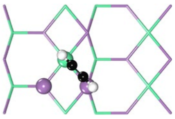
Side view	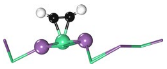	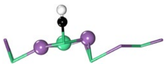	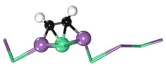	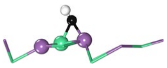	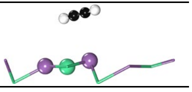
E_{ads} (eV)	-0.57	-0.41	-0.92	-0.76	-0.13
G_{ads} (eV)	-0.31	-0.16	-0.66	-0.49	0.16

Supplementary Table 6. Adsorption energies (E_{ads}/eV) and adsorption free energies (G_{ads}/eV) of C_2H_4 on the NiSb(101) surface.

Top view	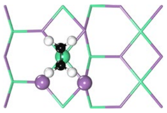	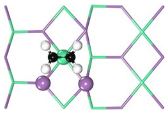	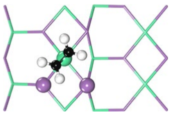	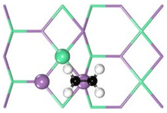	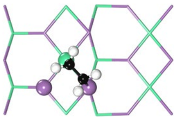
Side view	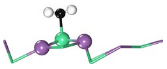	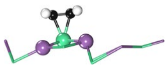	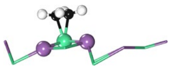	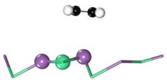	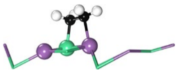
E_{ads} (eV)	-0.14	-0.30	-0.42	-0.14	-0.02
G_{ads} (eV)	0	-0.17	-0.29	0.02	0.08

Supplementary Table 7. Adsorption energies (E_{ads}/eV) and adsorption free energies (G_{ads}/eV) of C_2H_2 on the NiSb(102) surface.

Top view	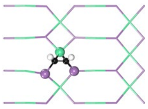	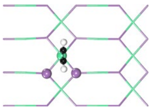	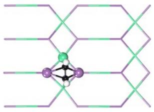	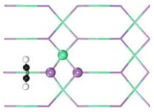	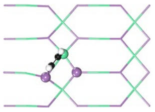
Side view	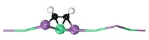	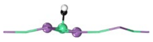	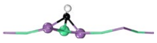	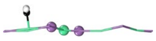	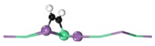
E_{ads} (eV)	-1.15	-0.43	-0.56	-0.82	-0.71
G_{ads} (eV)	-0.89	-0.17	-0.30	-0.56	-0.44

Supplementary Table 8. Adsorption energies (E_{ads}/eV) and adsorption free energies (G_{ads}/eV) of C_2H_4 on the NiSb(102) surface.

Top view	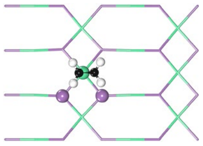	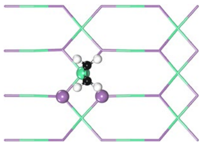	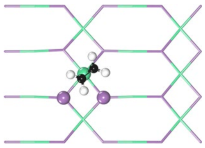	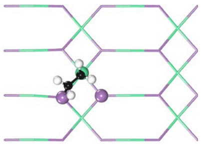
Side view	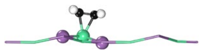	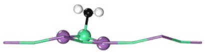	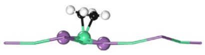	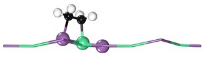
E_{ads} (eV)	-0.38	-0.26	-0.48	0.11
G_{ads} (eV)	-0.25	-0.13	-0.36	0.21

Supplementary Table 9. Adsorption energies (E_{ads}/eV) and adsorption free energies (G_{ads}/eV) of C_2H_2 on the NiSb(100) surface.

Top view	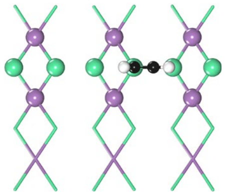	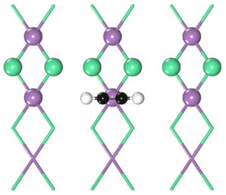	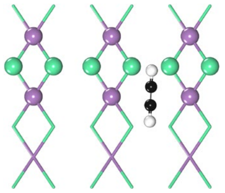
Side view	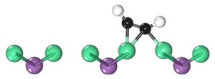	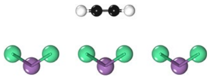	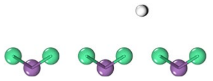
E_{ads} (eV)	-1.42	-0.18	-0.16
G_{ads} (eV)	-1.19	0.11	0.13

Supplementary Table 10. Adsorption energies (E_{ads}/eV) and adsorption free energies (G_{ads}/eV) of C_2H_4 on the NiSb(100) surface.

Top view	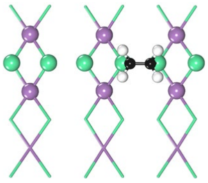	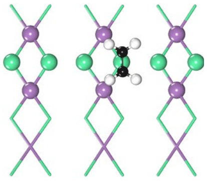	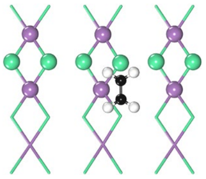	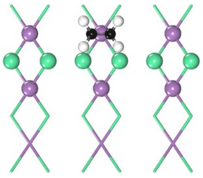
Side view	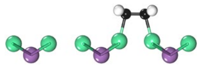	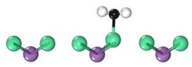	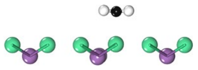	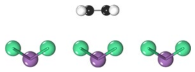
E_{ads} (eV)	-1.02	-0.96	-0.26	-0.18
G_{ads} (eV)	-0.91	-0.83	-0.12	-0.03

Supplementary Table 11. Properties of the Sb, Ni, NiSb and 2-NiSb catalysts.

**Sample**	**Ni loading (wt %)**	**Sb loading (wt %)**	**Mg loading (wt %)**	**Al loading (wt %)**	**Sb/Ni atomic ratio**	**BET surface area (m^2/g)**
Sb	0.27	0.44	35.57	17.15	/	126.47
Ni	14.00	0.33	26.37	12.60	/	135.35
NiSb	10.68	22.47	20.67	9.58	1.01	116.51
2-NiSb	10.77	24.07	20.69	8.82	1.07	111.44

Supplementary Table 12. Carbon balances at different acetylene conversion.

C_2H_2 conversion	Components	Ni			NiSb		
		Inlet (%)	Outlet (%)	Carbon balance (%)	Inlet (%)	Outlet (%)	Carbon balance (%)
Ca. 10%	C_2H_2	0.5000	0.4508		0.5000	0.4400	
	C_2H_4	20.0000	19.9205		20.0000	20.0500	
	C_2H_6	0.0000	0.0098		0.0000	0.0000	
	C_4	0.0000	0.0049		0.0000	0.0025	
	Total	20.5000	20.3909	99.47	20.5000	20.4950	99.98
		Inlet (%)	Outlet (%)	Carbon balance (%)	Inlet (%)	Outlet (%)	Carbon balance (%)
Ca. 90%	C_2H_2	0.5000	0.0400		0.5000	0.0425	
	C_2H_4	20.0000	18.7500		20.0000	20.4200	
	C_2H_6	0.0000	1.0125		0.0000	0.0046	
	C_4	0.0000	0.1250		0.0000	0.0088	
	Total	20.5000	20.0525	97.81	20.5000	20.4847	99.93

Supplementary References

1. Grimme S, Antony J, Ehrlich S, Krieg H. A consistent and accurate ab initio parametrization of density functional dispersion correction (DFT-D) for the 94 elements H-Pu. *J. Chem. Phys.* **132**, 154104 (2010).
2. Methfessel M, Paxton AT. High-precision sampling for Brillouin-zone integration in metals. *Phys Rev B Condens Matter* **40**, 3616-3621 (1989).
3. Cao Y*, et al.* Adsorption Site Regulation to Guide Atomic Design of Ni-Ga Catalysts for Acetylene Semi-Hydrogenation. *Angew. Chem. Int. Ed.* **59**, 11647-11652 (2020).
4. Millange F, Walton RI, O'Hare D. Time-resolved in situ X-ray diffraction study of the liquid-phase reconstruction of Mg-Al-carbonate hydrotalcite-like compounds. *J. Mater. Chem.* **10**, 1713-1720 (2000).
